# Prediction of Effective Drug Combinations by Chemical Interaction, Protein Interaction and Target Enrichment of KEGG Pathways

**DOI:** 10.1155/2013/723780

**Published:** 2013-09-05

**Authors:** Lei Chen, Bi-Qing Li, Ming-Yue Zheng, Jian Zhang, Kai-Yan Feng, Yu-Dong Cai

**Affiliations:** ^1^Institute of Systems Biology, Shanghai University, Shanghai 200444, China; ^2^College of Information Engineering, Shanghai Maritime University, Shanghai 201306, China; ^3^Key Laboratory of Systems Biology, Shanghai Institutes for Biological Sciences, Chinese Academy of Sciences, Shanghai 200031, China; ^4^State Key Laboratory of Drug Research, Shanghai Institute of Materia Medica, Shanghai 201203, China; ^5^Department of Ophthalmology, Shanghai First People's Hospital, School of Medicine, Shanghai Jiao Tong University, Shanghai 200080, China; ^6^Beijing Genomics Institute, Shenzhen Beishan Industrial Zone, Shenzhen 518083, China

## Abstract

Drug combinatorial therapy could be more effective in treating some complex diseases than single agents due to better efficacy and reduced side effects. Although some drug combinations are being used, their underlying molecular mechanisms are still poorly understood. Therefore, it is of great interest to deduce a novel drug combination by their molecular mechanisms in a robust and rigorous way. This paper attempts to predict effective drug combinations by a combined consideration of: (1) chemical interaction between drugs, (2) protein interactions between drugs' targets, and (3) target enrichment of KEGG pathways. A benchmark dataset was constructed, consisting of 121 confirmed effective combinations and 605 random combinations. Each drug combination was represented by 465 features derived from the aforementioned three properties. Some feature selection techniques, including Minimum Redundancy Maximum Relevance and Incremental Feature Selection, were adopted to extract the key features. Random forest model was built with its performance evaluated by 5-fold cross-validation. As a result, 55 key features providing the best prediction result were selected. These important features may help to gain insights into the mechanisms of drug combinations, and the proposed prediction model could become a useful tool for screening possible drug combinations.

## 1. Introduction

During the past decade, much effort has been spent on drug discovery, but the rate of new drug approvals is rather low. One of the reasons is that many of the human diseases are so complex with multiple targets that it is very difficult to design a single drug to hit all the targets. Since single targeted drugs can not treat these diseases very effectively [1], employing multiple targeted drugs is a favorable way, by which multiple target genes/proteins can be modulated simultaneously. It is already evidenced that drug combinations can improve therapeutic efficacy in many cases [2]. In addition, drug combinations may reduce toxicity and side effects that single targeted drugs may cause. Therefore, drug combinatorial therapy is considered to be effective in treating multifactorial complex diseases.

Drug combinations are becoming more and more popular nowadays, and they have been mainly discovered by experiments or clinical experience. On one hand, the molecular mechanisms of current drug combinations have not been clearly delineated; on the other, there are a myriad of possible drug combinations. Therefore, it is impractical to screen all possible combinations by conventional experiments or empirical rules. Computational methods may provide some valuable information and help to solve the problem. In recent years, some computational methods have been proposed to predict drug combinations [3–9]. However, these methods have not answered the question of which factors or features are more important for the determination of drug combinations, when it is essential to know which features and why they are able to distinguish good combinations from undesired ones. We propose a method here to identify the characteristic features of effective drug combinations, then analyze them and use them to predict novel combinations.

Drugs are combined according to their essential properties [10, 11]. In view of this, we considered the following three different kinds of properties: (1) chemical interactions between drugs in the combination [12], (2) protein interactions between the targets of drugs [13], and (3) target enrichment of KEGG pathways [14]. These properties were encoded into numeric digits, by which each drug combination was represented by a numeric vector. Feature selection methods, including minimum redundancy maximum relevance [15] and incremental feature selection, were adopted to extract key features. Random forest [16] was adopted as the classification model with its performance evaluated by 5-fold cross-validation. As a result, 55 key features, including one feature from chemical interaction, two features from protein interaction, and others from target enrichment of pathways, were identified and deemed as the most important features for the determination of effective drug combinations.

## 2. Materials and Methods

### 2.1. Benchmark Dataset

We retrieved all pairwise drug combinations from Zhao et al.'s study [8], which were parsed from FDA orange book [17], which lists approved drug products on the basis of safety and effectiveness by the Food and Drug Administration (FDA). The data in this book has been used as the object of study or reference in some studies [8, 18–21]. If the target information of any drug in the combination was not available, the combination it was involved in was excluded. As a result, 121 drug combinations were retrieved. These combinations were termed as “positive combinations”. Totally, 169 drugs were collected from the positive combinations, which were used to investigate drug combinations in this study.

There are 14,196 possible combinations among 169 drugs, where 121 combinations were solidly effective. For the other 14,075 combinations, their effects in treating diseases are not clear and which were assumed to be junk combinations. Among them we randomly selected 605 combinations as “negative combinations,” 5 times as many as the positive ones. The codes of positive and negative combinations can be found in Supplementary Material I (Supplementary Material available online at http://dx.doi.org/10.1155/2013/723780).

### 2.2. Drug Targets

It has been shown that the targets of agents are an important factor for the formation of effective drug combinations [9]. In this study, this information was also employed to construct classification features. The targets of 169 drugs were compiled from three drug target databases including KEGG (ftp://ftp.genome.jp/pub/kegg/medicus/drug/) [22], DrugBank [23], and Therapeutic Target Database (TTD) [24]. For each drug, the union of the targets from the three databases was regarded as the final target set. The codes of 169 drugs and their targets were available in Supplementary Material II.

### 2.3. Chemical-Chemical and Protein-Protein Interactions

It is based on the drugs and their targets to determine whether two drugs should be combined in usage. Thus, the interactions among drugs and among their targets are important for the determination of drug combinations. Here, the information of chemical-chemical interactions and protein-protein interactions were retrieved from Search Tool for Interactions of Chemicals (STITCH) [12] and Search Tool for the Retrieval of Interacting Genes/Proteins (STRING) [13], respectively, as the resources of gaining the classification features.

#### 2.3.1. Chemical-Chemical Interactions

The information of chemical-chemical interactions was downloaded from STITCH (http://stitch.embl.de/, “chemical_chemical.links.detailed.v3.0.tsv.gz”) [12]. Each interaction consists of two chemicals and five scores entitled “similarity,” “experimental,” “database,” “textmining,” and “combined score,” respectively. The score of “similarity” was obtained by combining open-source Chemistry Development Kit [25] to calculate chemical fingerprints and Tanimoto 2D chemical similarity scores [26, 27] between each pair of chemicals. The score of “experiment” was calculated according to the chemical's activities from MeSH pharmacological actions and NCI60 screens. The score of “Database” was calculated by the chemical reactions contained in pathway databases. The score of “textmining” was computed based on a cooccurrence scheme and a natural language processing (NLP) approach [28, 29]. The score of “combined score” was obtained by combining all of the information that was used to calculate the aforementioned four scores. Thus, the interactivity of two chemicals was determined by the last score. Since a larger score means that the corresponding chemicals can interact with high likelihood, the score is called confidence score in this study. For any two compounds *d*_1_ and *d*_2_, the confidence score of the interaction between them was denoted by *Q*_*c*_(*d*_1_, *d*_2_). Particularly, if the interaction between *d*_1_ and *d*_2_ is not available in STITCH, the confidence score of the interaction was set to zero, that is, *Q*_*c*_(*d*_1_, *d*_2_) = 0. 

#### 2.3.2. Protein-Protein Interactions

The file containing the information of protein-protein interactions was retrieved from STRING (http://string.embl.de/) [13]. The interactions in STRING include both physical and functional interactions. Like the chemical-chemical interaction in STITCH, each protein-protein interaction in STRING was labeled by a score integrating the information from experimental repositories, computational prediction methods, and public text collections [13]. Since the value of the score indicates the likelihood of occurrence of the interaction, it is also termed as confidence score. Here, let *Q*_*p*_(*p*_1_, *p*_2_) denote the interaction confidence score of the proteins *p*_1_ and *p*_2_. If *Q*_*p*_(*p*_1_, *p*_2_) > 0, we consider that proteins *p*_1_ and *p*_2_ are interactive proteins. Likewise, *Q*_*p*_(*p*_1_, *p*_2_) was set to zero if the interaction between *p*_1_ and *p*_2_  is not available in STRING.

### 2.4. Features of Drug Combinations

One of the most important steps of constructing a classification model is to encode each term by its essential properties. The definition of various features is described as follows, which can be deemed as important for the determination of drug combinations. For clarity, each drug combination was denoted by **D** = (*d*_1_, *d*_2_), where *d*_1_ and *d*_2_ are two drugs in the combination **D**, respectively.

We considered three aspects of drug combination: (1) chemical interaction between drugs, (2) protein interactions between drugs' targets, and (3) target enrichment of KEGG pathways. They reflect different levels of the drug-target relationship. The chemical interactions between drugs can indicate whether or not the drugs have antagonism. The protein interactions between drugs' targets and the KEGG enrichment scores of drugs' targets represent the biological functions that the drugs can perturb.

#### 2.4.1. Chemical Interaction

Two drugs forming a solid combination are more likely to have similar properties. Hence, the interactive chemicals defined in Section 2.3 can share similar biological functions [30, 31] with high probability. Accordingly, the interaction confidence score of two drugs in the combination **D**, that is, *Q*_*c*_(*d*_1_, *d*_2_), was taken as a feature. 

#### 2.4.2. Protein Interaction

Since drugs take their effects by hitting some target proteins, the target proteins of two drugs are related to each other in a special way [9]. In addition, the interactive proteins defined in Section 2.3 always share similar functions [32, 33]. Thus, it is a reasonable scheme using the information of the protein-protein interactions retrieved from STRING to indicate the special relationship between drug target proteins. For drug combination **D** = (*d*_1_, *d*_2_), their targets were formulated as *T*(*d*_1_) = {*T*_1_^1^, *T*_1_^2^,…, *T*_1_^*n*^} and *T*(*d*_2_) = {*T*_2_^1^, *T*_2_^2^,…, *T*_2_^*m*^}, respectively. We defined the following two kinds of features to describe their relationship.

(1) Protein interactions between the target groups: for any protein *T*_1_^*i*^ in *T*(*d*_1_) and any protein *T*_2_^*j*^ in *T*(*d*_2_), their interaction confidence score can be obtained from STRING [13] (see Section 2.3). The maximum and mean values of these scores were formulated as follows:
  (1) $$\begin{array}{c} \mathrm{Max}\left\{Q_{p}\left(T_{1}^{i},T_{2}^{j}\right):1\leq i\leq n,1\leq j\leq m\right\},\\ \text{Mean}\left\{Q_{p}\left(T_{1}^{i},T_{2}^{j}\right):1\leq i\leq n,1\leq j\leq m\right\}, \end{array}$$
  which were taken as features.
(2) Protein interactions inside the target groups: for drug *d*_*i*_, we can obtain two values *v*_*i*_^1^ and *v*_*i*_^2^, where *v*_*i*_^1^ and *v*_*i*_^2^ are the maximum value and mean value of interaction confidence scores between target proteins in *T*(*d*_*i*_), respectively. Since there is no order in the information for a drug combination, (*d*_1_, *d*_2_) and (*d*_2_, *d*_1_) are equivalent. In view of this, we refined *v*_1_^1^, *v*_1_^2^, *v*_2_^1^, and *v*_2_^2^ as follows:
  (2) $$\begin{array}{c} v_{1}^{1}+v_{2}^{1},v_{1}^{2}+v_{2}^{2},\left|v_{1}^{1}-v_{2}^{1}\right|,\left|v_{1}^{2}-v_{2}^{2}\right|, \end{array}$$
  which were also taken as features in the study.

#### 2.4.3. Target Enrichment for KEGG Pathway

The target proteins of a drug are distributed in many pathways, that is, a single drug may belong to multiple pathways and modulate their functions. To partially account for this effect, we employed the pathways in KEGG [22] and KEGG enrichment score [14, 34, 35] to quantify the relation between drugs and pathways in KEGG. For drug *d*_*i*_ and KEGG pathway *P*_*j*_, the KEGG enrichment score is defined as the −log⁡_10_ of the hypergeometric test *p* value of gene set *G*_*i*_, which includes targets of drug *d*_*i*_ and their direct neighbors in STRING network. It can be calculated as follows:

(3) $$\begin{array}{c} {\text{Score}}_{i}^{j}=-\log_{10}\left(\sum_{k=m}^{n}\frac{\left(\begin{array}{c} M\\ k \end{array}\right)\left(\begin{array}{c} N-M\\ n-k \end{array}\right)}{\left(\begin{array}{c} N\\ n \end{array}\right)}\right), \end{array}$$

where *N* is the number of genes in human, *M* is the number of genes annotated to the KEGG pathway *P*_*j*_, *n* is the number of genes in gene set *G*_*i*_, and *m* is the number of genes both in gene set *G*_*i*_ and in KEGG pathway *P*_*j*_. The KEGG enrichment scores can measure the biological functions of the genes. The higher enrichment score indicates that this gene is more likely to have this function. Unlike traditional binary function annotation in which if it is annotated, it is one; otherwise, it is zero, the KEGG enrichment gives a probability of this gene that has this function by considering its microenvironment on the protein-protein interaction network. If drug targets are more represented in one pathway, the enrichment score of this pathway will be greater. There were 229 KEGG enrichment scores for each drug *d*_*i*_  (*i* = 1,2) in a drug combination **D** denoted by *e*_*i*_^1^, *e*_*i*_^2^,…, *e*_*i*_^229^  (*i* = 1,2). Similar to the features of protein interactions, 458 features can be derived from these enrichment scores as follows:

(4) $$\begin{array}{c} e_{1}^{1}+e_{2}^{1},e_{1}^{2}+e_{2}^{2},\ldots ,e_{1}^{229}+e_{2}^{229}, \end{array}$$

(5) $$\begin{array}{c} \left|e_{1}^{1}-e_{2}^{1}\right|,\left|e_{1}^{2}-e_{2}^{2}\right|,\ldots ,\left|e_{1}^{229}-e_{2}^{229}\right|. \end{array}$$

In summary, there were one feature from chemical interaction, six features from protein interaction, and 458 features from target enrichment, totally (1 + 6 + 458) = 465 features. Thus, each drug combination can be represented by a vector in a 465 D (dimensional) space, that is, each feature is deemed as a dimension.

### 2.5. Random Forest

Random forest, developed by Breiman [16], is an ensemble classifier integrating multiple decision trees. The procedure of constructing each decision tree is briefly described as follows.

- Let *N* be the number of training samples. We randomly take *N* samples from the training samples, but with replacement from the original data, to construct the decision tree, while the rest of the samples are used to evaluate the error of the tree by predicting their classes.
- Let *M* be the total number of features. *m* is a positive integer that is much less than *M*. When constructing the tree, *m* features are selected randomly from *M* features at each node, and the most optimized split on these *m* features is utilized to split the node.
- Each tree is fully grown without pruning.

For a query sample, each decision tree would make a prediction and the overall prediction is decided by voting.

Weka 3.6.4 [36] is a software collecting various state-of-art machine learning algorithms. Random forest is implemented by a classifier named RandomForest in Weka, which was adopted as the classification model and run with its default parameters in the study. In its default configuration, each random forest consists of 10 decision trees, and *m* in step (II) is set to [log⁡_2_⁡*M* + 1], that is, *m* = [log⁡_2_⁡*M* + 1]. For a query drug combination, each of 10 decision trees would give its prediction (“positive” or “negative”). Then, the final predicted result is the class (“positive” or “negative”) obtaining a majority vote.

### 2.6. Accuracy Measurement

For a two-class classification problem, there are four entries in the confusion matrix: TP, TN, FP, and FN, where TP represents true positives, TN true negatives, FP false positives, and FN false negatives [37, 38]. Based on these values, the prediction accuracy (ACC), specificity (SP), sensitivity (SN), Matthews's correlation coefficient (MCC) [39], and Area Under ROC curve (AUC) score [40] are often used to evaluate the performance of the classification model. They can be calculated as follows:

(6) $$\begin{array}{c} \text{ACC}=\frac{\text{TP}+\text{TN}}{\text{TP}+\text{TN}+\text{FP}+\text{FN}},\\ \text{SP}=\frac{\text{TN}}{\text{TN}+\text{FP}},\\ \text{SN}=\frac{\text{TP}}{\text{TP}+\text{FN}},\\ \text{MCC}=\frac{\text{TP}\cdot \text{TN}-\text{FP}\cdot \text{FN}}{\sqrt{\left(\text{TN}+\text{FN}\right)\cdot \left(\text{TN}+\text{FP}\right)\cdot \left(\text{TP}+\text{FN}\right)\cdot \left(\text{TP}+\text{FP}\right)}}. \end{array}$$

MCC is a measure of the quality of classifiers on the whole and is deemed to be a balanced measure even if the classes are of very different sizes. Thus, it has been widely used to evaluate the quality of classifiers proposed in many studies [14, 37, 41–44]. AUC score is another measurement to evaluate the performance of the classification model on the whole other than MCC. It is the normalized area under the ROC curve, which is plotted in the coordinate system with sensitivity as *Y*-axis and 1 − specificity (calculated by FP/(FP + TN)) as *X*-axis under various classification thresholds. In this study, we selected MCC to measure the performance of the method on the whole, while AUC score was also provided for reference.

### 2.7. 5-Fold Cross-Validation

5-fold cross-validation is often used to evaluate the performance of various classification models [45]. In 5-fold cross-validation, the original dataset is equally separated into five portions at random. Each portion is used as testing data in turn and the remaining 4 portions are used as training data. Thus, each datum is tested exactly once since each portion is tested exactly once during the procedure. In the study, 5-fold cross-validation was adopted to evaluate the model presented.

### 2.8. Minimum Redundancy Maximum Relevance (mRMR)

As described in Section 2.4, each drug combination was represented by various features. However, not all features contribute to the classification. In view of this, it is necessary to employ feature selection techniques to analyze these features and extract the useful ones. Minimum redundancy maximum relevance was proposed by Peng et al. [15], and it is deemed as an outstanding method for extracting important information from complicated systems [46–49], which was also adopted in the study. We could obtain two lists by mRMR program: MaxRel features list and mRMR features list, where the MaxRel features list sorts the features according to the criterion that features contributing more to the classification will have higher ranks, while mRMR features list is produced according to the criteria of both MaxRel and minimum redundancy, which ensures that a feature having minimum redundancy among the already selected features and giving the most contribution to the classification will tend to have a higher rank. The MaxRel features list and mRMR features list were formulated as follows:

(7) $$\begin{array}{c} \text{MaxRel}\;\;\text{features}\;\;\text{list}:F_{M}=\left[f_{1}^{M},f_{2}^{M},\ldots ,f_{N}^{M}\right],\\ \text{mRMR}\;\;\text{features}\;\;\text{list}:F_{m}=\left[f_{1}^{m},f_{2}^{m},\ldots ,f_{N}^{m}\right], \end{array}$$

where *N* represents the total number of features. For detailed description of mRMR method and its analysis, please refer to Peng et al.'s paper [15].

### 2.9. Incremental Feature Selection (IFS)

Based on mRMR features list *F*_*m*_ = [*f*_1_^*m*^, *f*_2_^*m*^,…, *f*_*N*_^*m*^], incremental feature selection was performed as follows:

- construct *N* feature subsets, in a way that the *i*th feature subset is defined as *F*_*m*_^*i*^ = [*f*_1_^*m*^, *f*_2_^*m*^,…, *f*_*i*_^*m*^]  (1 ≤ *i* ≤ *N*);
- for each *i*  (1 ≤ *i* ≤ *N*), execute RandomForest in Weka using features in *F*_*m*_^*i*^, respectively, evaluated by 5-fold cross-validation, thereby obtaining ACC, SP, SN, MCC, and AUC scores as described in Section 2.6;
- plot an IFS curve with MCC value as its *Y*-axis and the superscript *i* of *F*_*m*_^*i*^ as its *X*-axis.

## 3. Results and Discussion

### 3.1. mRMR Results

The mRMR program was downloaded from the website http://research.janelia.org/peng/proj/mRMR/ and it was executed with its default parameters. As described in Section 2.8, we can obtain two feature lists: MaxRel features list and mRMR features list (available as Supplementary Material III). The ranks of features in MaxRel features list reflect their contribution to classification. Here, we investigated the first 10 features in this list (see the first table in Supplementary Material III for details). The first feature (“F1”) is the interaction confidence score of *d*_1_ and *d*_2_ in the combination **D** = (*d*_1_, *d*_2_) and the second feature (“F2”) is the maximum confidence score between the targets of drug *d*_1_ and *d*_2_, indicating that the interactions of drugs and their targets are key factors for the determination of drug combinations. The later one is partially consistent with the previous results [9]. The remaining 8 features are related to the following seven pathways: (I) hsa04964 (“Proximal tubule bicarbonate reclamation”), (II) hsa00052 (“galactose metabolism”), (III) hsa04970 (“salivary secretion”), (IV) hsa00910 (“nitrogen metabolism”), (V) hsa05215 (“prostate cancer”), (VI) hsa05130 (“pathogenic Escherichia coli infection”), and (VII) hsa00520 (“amino sugar and nucleotide sugar metabolism”), where pathway hsa00910 (“nitrogen metabolism”) involved two features, while the others involved one feature.

### 3.2. IFS Results

**Figure 1:**
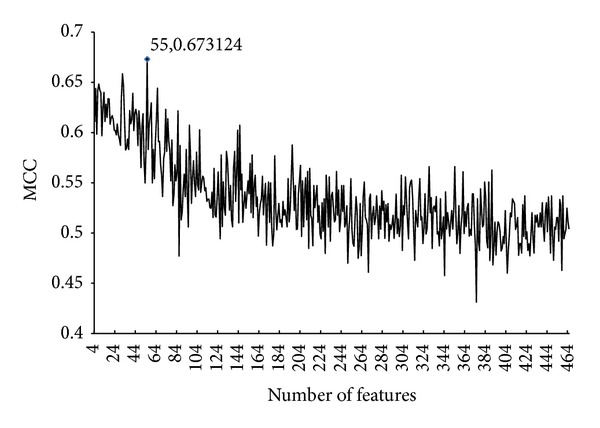
The IFS curve. The *X*-axis represents the number of features participating in the classification model. The *Y*-axis represents the Matthews's correlation coefficient (MCC) value evaluated by the classification model and 5-fold cross-validation. The highest MCC value of IFS is 0.6731 using 55 features.

**Figure 2:**
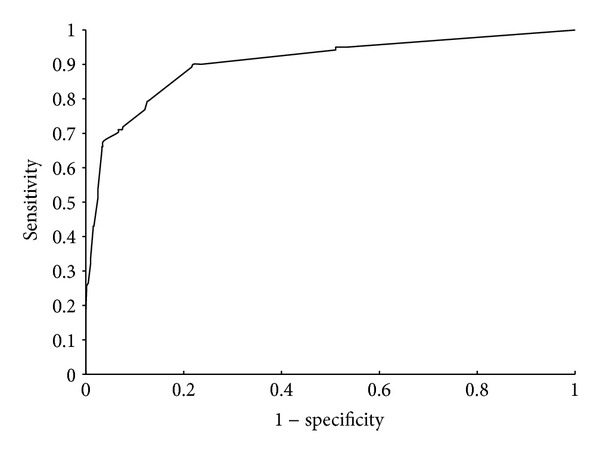
ROC curve. The curve was obtained by the classification model using first 55 features in mRMR features list. The *X*-axis and *Y*-axis of each point in the curve represent the 1 − specificity and sensitivity, respectively, under a certain classification threshold.

Shown in Figure 1 is the IFS curve with MCC value, predicted by RandomForest in Weka and evaluated by 5-fold cross-validation, which takes MCC as its *Y*-axis and the number of features participating in the classification model as its *X*-axis. For the detailed IFS data, please refer to Supplementary Material IV. It is observed that the highest MCC value is 0.6731, obtained when the first 55 features were used in the mRMR features list (see the second table in Supplementary Material III for details). The prediction accuracy (ACC), specificity (SP), and sensitivity (SN) are 0.9146, 0.9669, and 0.6529, respectively. Furthermore, AUC score obtained by the classification model using these 55 features was 0.8803, indicating that this model has good discriminating power for drug combinations. Its related ROC curve is shown in Figure 2. These 55 features were deemed as the optimal features for the determination of drug combinations, composing the optimal feature set **OS**, that is, **O** **S** = *F*_*m*_^55^. In **OS**, three features were from chemical and protein interactions. In details, besides “F1” and “F2” in Section 3.1, “F3”, with the rank of 25 in **OS**, is the mean value of confidence scores between the targets of drug *d*_1_ and the targets of *d*_2_. The rest 52 features were related to 50 pathways (see Table 1 for details), where the pathway hsa04964 (“proximal tubule bicarbonate reclamation”) and hsa05020 (“prion diseases”) involved two features, respectively, while the other pathways involved exactly one feature. Among the 52 features, 36 were obtained by (5), while the rest 16 by (4) (cf. Table 1). It is clear that the features obtained by (5), measuring the difference of enrichment scores, were better discriminators than those obtained by (4), measuring the sum of enrichment scores. It is suggested that in a drug combination, the targets of two drugs should relate to each other in a special way. 

### 3.3. Analysis of Optimal Features

First, we find that there are 8 mRMR features among the top ten features in the MaxRel list mentioned in Section 3.1. It is suggested that these 8 features are particularly good at distinguishing drug pairs.

It is not surprising that the first feature is “F1” (Supplementary Material III), which is the confidence score of interaction between two drugs. The key assumption underlying most drug prediction algorithms is that similar drugs have a tendency to share similar targets [50]. This has been observed due to chemical similarity [26, 51]. In addition, it has been proved that interactive chemicals are more likely to share similar biological functions [30, 31].

The second optimal feature is the absolute difference in the value of two drugs' enrichment score in prostate cancer pathway (“abs(hsa05215_1-hsa05215_2),” refer to Supplementary Material III). The prostate cancer pathway is mainly characterized by key molecular changes in prostate cancer cells including cell cycle, carcinogen defenses, cell adhesion, migration and growth, and androgens [52], which are involved in numerous cancers. Therefore, lots of antineoplastic drugs are designed targeting genes in this pathway. In the study of Wedel et al. [53], they proposed a triple drug combination including RAD001, AEE788, and VPA, which represented a stronger anticancer effect than any single drug. Notably, cyclin B, cdk1, 2, and 4 were reduced, since strong antitumor properties related to adhesion dynamics and cell growth became visible. Therefore, this triple drug combination might possess the potential in the treatment of advanced prostate cancer as well as other cancers [53]. In addition, it has been reported that drug combination can extend life for men with prostate cancer [54]. Furthermore, Danquah et al. have revealed that a treatment strategy with novel drug combination is a promising approach to treat androgen-independent prostate cancer [55]. Overall, the genes in prostate cancer pathway may provide clues for antineoplastic drugs design and application of drug combinations.

Drug combination approaches are especially applicable to cancer treatment. On one hand, most tumors depend on more than one signaling pathways for their growth, survival, invasion, and metastasis; on the other, multiple cell signaling pathways may control a single step of tumorigenesis. Thus, efficacious and durable responses in cancer may require a combined usage of conventional single-targeted agents [56]. Moreover, cells may develop drug-resistant mutations to a single-targeted agent and most cancers have four to seven independent mutations [57]. The chance of overcoming such resistance can be significantly increased by using agent or drug that inhibits multiple pathways or their combination [58–60].

The third critical feature for drug combination determination is the sum of enrichment score of two drugs in proximal tubule bicarbonate reclamation pathway (“hsa04964_1+hsa04964_2,” refer to Supplementary Material III). It has been reported that primary porcine proximal tubular cells play an important role in transepithelial drug transport in human kidney [61]. Many genes in this pathway have been proved to be related to drug response, drug toxicity, and drug transport. CA4 (carbonic anhydrase IV) is a member of carbonic anhydrases (CAs) family, which is a group of universally expressed metalloenzymes related to multiple pathological and physiological processes, such as lipogenesis, gluconeogenesis, tumorigenicity, ureagenesis, and the virulence and growth of various pathogens [62]. Apart from the already known roles of CA inhibitors (CAIs) as antiglaucoma and diuretics drugs, CAIs could also possess the potential to be novel anticancer, anti-infective, and antiobesity drugs [62]. PCK1 (phosphoenolpyruvate carboxykinase 1) is a key control point during the regulation of gluconeogenesis. It has been shown that PCK1 is involved in the processes of small molecule biochemistry, carbohydrate metabolism, molecular transport, and response to drugs including 5-tert-butyl-3H-1,2-dithiole-3-thione (TBD), 3H-1,2-dithiole-3-thione (D3T), and its analogues 4-methyl-5-pyrazinyl-3H-1,2-dithiole-3-thione (OLT) [63]. MDH1 (malate dehydrogenase 1) in this pathway has been reported to be relevant with drug toxicity [64, 65]. Another gene in this pathway worth mentioning is AQP1 (Aquaporin-1), which was highly expressed in endothelial cell membranes and involved in water transfer across or into these cells. It has been reported that AQP1 plays a role in response to acetazolamide [66] and drug transport [67, 68]. In addition, ATP1B1 (Na(+)-K(+) ATPaseB1) in this pathway has also been revealed to be related to drug response in breast cancer cell lines [69]. 

The fourth feature in the optimal feature set is “F2”, which is the maximum confidence score between targets of two drugs in a drug combination. Since drugs sharing the same targets usually have similar pharmacology, they are likely to be replaceable with each other when combined with another drug for similar purposes [8]. In general, drugs are combined according to the mechanisms of action, which is characterized by the properties of drugs including their pharmacology and targets [10, 11]. Therefore, drugs in a drug combination have a high tendency to target the same proteins or similar pharmacology [70].

Besides the top four optimal features, there are several other critical pathways in the optimal feature set. It has been shown that the steroid hormone biosynthesis pathway (hsa00140) can act as a target for endocrine-disrupting chemicals [71], and inhibitors of steroidal cytochrome P450 enzymes have the potential to be targets for drug development [72]. Staphylococcus aureus infection pathway (hsa05150) has been shown to be related to drug resistance [73, 74]. A large amount of studies have shown that the hedgehog signaling pathway (hsa04340) has the potential to be a target for anticancer drug discovery [75]. In addition, the inhibition of hedgehog signaling can enhance the delivery of chemotherapy in a mouse model of pancreatic cancer [76]. Furthermore, hedgehog signaling can regulate the drug sensitivity by targeting ABC transporters in epithelial ovarian cancer (EOC) [77]. It has been proposed that glycosaminoglycan degradation pathway (hsa00531) has significant therapeutic value in cancer [78]. Because dysregulated glycosaminoglycan degradation plays an important role in tumorigenesis, targeting glycosaminoglycan-degrading enzymes is a promising anticancer strategy. Dysregulated expression of glycosaminoglycans is ubiquitous in cancer and has been shown to associate with clinical prognosis in several malignant neoplasms. Recently, research on the biological functions of these molecules in tumor angiogenesis, tumor metastasis, and cancer biology has facilitated the development of drugs targeting them. In addition, glycosaminoglycans are utilized as tumor-specific targeting vehicles and delivery for chemotherapeutics and toxins. Animal studies as well as clinical trials have shown the clinical relevance of glycosaminoglycan-based drugs and the utility of glycosaminoglycans as therapeutic targets [78]. Another noteworthy pathway is carbohydrate digestion and absorption pathway (hsa04973). Genes in this pathway have been widely used as antidiabetic drugs target [79, 80].

## 4. Conclusions

In this study, we analyzed molecular mechanisms of drug combinations by extracting certain kinds of features from each combination. After adopting Minimum Redundancy Maximum Relevance and Incremental Feature Selection as the feature selection techniques and random forest as the classification model, 55 optimal features were obtained, with which the classification model achieved the best performance. The results show that the chemical interaction between drugs in the combination and protein interactions between their targets are important for the determination of drug combinations. In addition, some KEGG pathways important for screening drug combinations are also highlighted. We hope that this contribution may help to screen new drug combinations.

## Supplementary Material

The Supplementary Material contains four files. In details, Supplementary Material I lists 726 drug compounds investigated in this study; Supplementary Material II lists the targets of 169 drugs; Supplementary Material III lists the results obtained by mRMR method; Supplementary Material IV lists the accuracies obtained by IFS and random forest.

## Figures and Tables

**Table 1:** The 48 pathways related to features in the optimal feature set.

Index	Pathway ID and name	The rank of related features (+/−)^a^
1	hsa05215 prostate cancer	2 (−)
2	hsa04964 proximal tubule bicarbonate reclamation	3 (+), 48 (−)
3	hsa00140 steroid hormone biosynthesis	5 (−)
4	hsa04145 phagosome	6 (+)
5	hsa05150 staphylococcus aureus infection	7 (−)
6	hsa04973 carbohydrate digestion and absorption	8 (−)
7	hsa04340 hedgehog signaling pathway	9 (−)
8	hsa00052 galactose metabolism	10 (+)
9	hsa04310 wnt signaling pathway	11 (−)
10	hsa00531 glycosaminoglycan degradation	12 (+)
11	hsa04972 pancreatic secretion	13 (+)
12	hsa04976 bile secretion	14 (−)
13	hsa03018 rNA degradation	15 (−)
14	hsa04744 phototransduction	16 (−)
15	hsa04977 vitamin digestion and absorption	17 (−)
16	hsa04330 notch signaling pathway	18 (−)
17	hsa00430 taurine and hypotaurine metabolism	19 (−)
18	hsa05130 pathogenic Escherichia coli infection	20 (−)
19	hsa00920 sulfur metabolism	21 (+)
20	hsa00785 lipoic acid metabolism	22 (−)
21	hsa05020 prion diseases	23 (+), 54 (−)
22	hsa00511 other glycan degradation	24 (+)
23	hsa04320 dorso-ventral axis formation	26 (−)
24	hsa00520 amino sugar and nucleotide sugar metabolism	27 (−)
25	hsa00310 lysine degradation	28 (−)
26	hsa00270 cysteine and methionine metabolism	29 (−)
27	hsa04115 p53 signaling pathway	30 (−)
28	hsa04966 collecting duct acid secretion	31 (+)
29	hsa00830 retinol metabolism	32 (−)
30	hsa00910 nitrogen metabolism	33 (−)
31	hsa05217 basal cell carcinoma	34 (−)
32	hsa05010 alzheimer's disease	35 (−)
33	hsa04150 mTOR signaling pathway	36 (−)
34	hsa00532 glycosaminoglycan biosynthesis chondroitin sulfate	37 (+)
35	hsa04514 cell adhesion molecules (CAMs)	38 (−)
36	hsa04975 fat digestion and absorption	39 (−)
37	hsa05110 vibrio cholerae infection	40 (+)
38	hsa05416 viral myocarditis	41 (−)
39	hsa05012 parkinson's disease	42 (−)
40	hsa04614 renin-angiotensin system	43 (−)
41	hsa04130 SNARE interactions in vesicular transport	44 (+)
42	hsa00480 glutathione metabolism	45 (+)
43	hsa05211 renal cell carcinoma	46 (+)
44	hsa05322 systemic lupus erythematosus	47 (−)
45	hsa04120 ubiquitin mediated proteolysis	49 (+)
46	hsa00780 biotin metabolism	50 (+)
47	hsa00630 glyoxylate and dicarboxylate metabolism	51 (−)
48	hsa00510 n-glycan biosynthesis	52 (−)
49	hsa00061 fatty acid biosynthesis	53 (−)
50	hsa00232 caffeine metabolism	55 (−)

a: “+” and “−” in this column indicate that the feature is related to the pathways obtained by (4) and (5), respectively. For example, the feature in the first row with “−” was calculated as abs(hsa05215_1-hsa05215_2).
