# Supplementary material for: Prediction of Effective Drug Combinations by Chemical Interaction, Protein Interaction and Target Enrichment of KEGG Pathways
Source: Biomed Res Int. 2013 Sep 5;2013:723780. doi: 10.1155/2013/723780 (PMC3780555; doi:10.1155/2013/723780)
Supplement: Supplementary file 1 — The Supplementary Material contains four files. In details, Supplementary Material I lists 726 drug compounds investigated in this study; Supplementary Material II lists the targets of 169 drugs; Supplementary Material III lists the results obtained by mRMR method; Supplementary Material IV lists the accuracies obtained by IFS and random forest. [file 723780.f1.pdf]

Supplementary Material I: The benchmark dataset consisted of 726 combinations, of which 121 were positive combinations and 605 were negative combinations.

Index	Drug #1	Drug #2	Positive or Negative
1	D00236	D02487	Positive
2	D00475	D00570	Positive
3	D00848	D02272	Positive
4	D00217	D03182	Positive
5	D00340	D00626	Positive
6	D00023	D00165	Positive
7	D01024	D03820	Positive
8	D00251	D00340	Positive
9	D00554	D01294	Positive
10	D00340	D00627	Positive
11	D00683	D02212	Positive
12	D00593	D00945	Positive
13	D00432	D00650	Positive
14	D00485	D00970	Positive
15	D00340	D00400	Positive
16	D00340	D00362	Positive
17	D00554	D00950	Positive
18	D00267	D00809	Positive
19	D00126	D00847	Positive
20	D00615	D06412	Positive
21	D01450	D02149	Positive
22	D00455	D01203	Positive
23	D00454	D00823	Positive
24	D00358	D00551	Positive
25	D00340	D01204	Positive
26	D00340	D02082	Positive
27	D00679	D01453	Positive
28	D00113	D00469	Positive
29	D00944	D08996	Positive
30	D00340	D01302	Positive
31	D00842	D02095	Positive
32	D01413	D03799	Positive
33	D00105	D00953	Positive
34	D00126	D00511	Positive
35	D00217	D00847	Positive
36	D00554	D03917	Positive
37	D00400	D06412	Positive
38	D00516	D01302	Positive

39	D00480	D02101	Positive
40	D00554	D00953	Positive
41	D00615	D00887	Positive
42	D00603	D02076	Positive
43	D00340	D00459	Positive
44	D00335	D00944	Positive
45	D00340	D00357	Positive
46	D00480	D00848	Positive
47	D00182	D00575	Positive
48	D00109	D00847	Positive
49	D00088	D02216	Positive
50	D00615	D00627	Positive
51	D00554	D05205	Positive
52	D00336	D00944	Positive
53	D00554	D04104	Positive
54	D00235	D00272	Positive
55	D00272	D00604	Positive
56	D00503	D00809	Positive
57	D00049	D00434	Positive
58	D00211	D00346	Positive
59	D00095	D02086	Positive
60	D00217	D02101	Positive
61	D00246	D05277	Positive
62	D00186	D02216	Positive
63	D01066	D03048	Positive
64	D00687	D01708	Positive
65	D00554	D02367	Positive
66	D00340	D00523	Positive
67	D00944	D06645	Positive
68	D00182	D00554	Positive
69	D00340	D00634	Positive
70	D00340	D00623	Positive
71	D00105	D00950	Positive
72	D00603	D00653	Positive
73	D01066	D06236	Positive
74	D00554	D05209	Positive
75	D00088	D00222	Positive
76	D00105	D03917	Positive
77	D00340	D00405	Positive
78	D00594	D00944	Positive
79	D00340	D00386	Positive
80	D00593	D00596	Positive
81	D00511	D01002	Positive

82	D00340	D00483	Positive
83	D00118	D01984	Positive
84	D01340	D02227	Positive
85	D00738	D02388	Positive
86	D00340	D06412	Positive
87	D00059	D00558	Positive
88	D00615	D01204	Positive
89	D00383	D00619	Positive
90	D00109	D00302	Positive
91	D00690	D01373	Positive
92	D00358	D00553	Positive
93	D00596	D00944	Positive
94	D00217	D00482	Positive
95	D00340	D00443	Positive
96	D00217	D02227	Positive
97	D00400	D00615	Positive
98	D00480	D00511	Positive
99	D00972	D01402	Positive
100	D00126	D00485	Positive
101	D00282	D01637	Positive
102	D00105	D05209	Positive
103	D00615	D00620	Positive
104	D02069	D03860	Positive
105	D00419	D00904	Positive
106	D00944	D00945	Positive
107	D07132	D08249	Positive
108	D00217	D01355	Positive
109	D00186	D00292	Positive
110	D00434	D01966	Positive
111	D00665	D05649	Positive
112	D00109	D00402	Positive
113	D00340	D00649	Positive
114	D00340	D00620	Positive
115	D00188	D00939	Positive
116	D00994	D02069	Positive
117	D00676	D00970	Positive
118	D00049	D00359	Positive
119	D00340	D00621	Positive
120	D00340	D07992	Positive
121	D00217	D05632	Positive
122	D00023	D00217	Negative
123	D00023	D00335	Negative
124	D00023	D00848	Negative

125	D00023	D01002	Negative
126	D00023	D01966	Negative
127	D00023	D02388	Negative
128	D00049	D00634	Negative
129	D00049	D00665	Negative
130	D00049	D00676	Negative
131	D00049	D01302	Negative
132	D00049	D02082	Negative
133	D00049	D03182	Negative
134	D00059	D00236	Negative
135	D00059	D00282	Negative
136	D00059	D00480	Negative
137	D00059	D00553	Negative
138	D00059	D00679	Negative
139	D00059	D00729	Negative
140	D00059	D00945	Negative
141	D00059	D02076	Negative
142	D00059	D02483	Negative
143	D00068	D00145	Negative
144	D00068	D00292	Negative
145	D00068	D00346	Negative
146	D00068	D00604	Negative
147	D00068	D00743	Negative
148	D00068	D00823	Negative
149	D00068	D02101	Negative
150	D00068	D03799	Negative
151	D00068	D05649	Negative
152	D00088	D00626	Negative
153	D00088	D00665	Negative
154	D00088	D00944	Negative
155	D00088	D06412	Negative
156	D00094	D00402	Negative
157	D00094	D00419	Negative
158	D00094	D00945	Negative
159	D00094	D01302	Negative
160	D00094	D01413	Negative
161	D00095	D00386	Negative
162	D00095	D00649	Negative
163	D00095	D00809	Negative
164	D00095	D01966	Negative
165	D00105	D00113	Negative
166	D00105	D00251	Negative
167	D00105	D00432	Negative

168	D00105	D00939	Negative
169	D00105	D01413	Negative
170	D00105	D02060	Negative
171	D00105	D02149	Negative
172	D00109	D00419	Negative
173	D00109	D00443	Negative
174	D00109	D00554	Negative
175	D00109	D00981	Negative
176	D00109	D01450	Negative
177	D00109	D01708	Negative
178	D00109	D02272	Negative
179	D00109	D06236	Negative
180	D00109	D08127	Negative
181	D00113	D00397	Negative
182	D00113	D00604	Negative
183	D00113	D01002	Negative
184	D00113	D01066	Negative
185	D00113	D02149	Negative
186	D00113	D02216	Negative
187	D00113	D02487	Negative
188	D00118	D00246	Negative
189	D00118	D00434	Negative
190	D00118	D00980	Negative
191	D00118	D05632	Negative
192	D00118	D08127	Negative
193	D00126	D00165	Negative
194	D00126	D00282	Negative
195	D00126	D00405	Negative
196	D00126	D00475	Negative
197	D00126	D00621	Negative
198	D00126	D00904	Negative
199	D00145	D00362	Negative
200	D00145	D00683	Negative
201	D00145	D00823	Negative
202	D00145	D01413	Negative
203	D00145	D01450	Negative
204	D00145	D05632	Negative
205	D00145	D08996	Negative
206	D00165	D00182	Negative
207	D00165	D00222	Negative
208	D00165	D00383	Negative
209	D00165	D01112	Negative
210	D00165	D01204	Negative

211	D00165	D02095	Negative
212	D00165	D02272	Negative
213	D00165	D05649	Negative
214	D00182	D00405	Negative
215	D00182	D01204	Negative
216	D00182	D03799	Negative
217	D00182	D04104	Negative
218	D00182	D05632	Negative
219	D00186	D00246	Negative
220	D00186	D00397	Negative
221	D00186	D00596	Negative
222	D00186	D00634	Negative
223	D00186	D00687	Negative
224	D00186	D00823	Negative
225	D00186	D00983	Negative
226	D00186	D01373	Negative
227	D00186	D01450	Negative
228	D00186	D01637	Negative
229	D00186	D02149	Negative
230	D00186	D02272	Negative
231	D00186	D02487	Negative
232	D00186	D03048	Negative
233	D00186	D05277	Negative
234	D00186	D08249	Negative
235	D00188	D00397	Negative
236	D00188	D00729	Negative
237	D00188	D00848	Negative
238	D00188	D00972	Negative
239	D00188	D01413	Negative
240	D00188	D03917	Negative
241	D00211	D00336	Negative
242	D00211	D00523	Negative
243	D00211	D00554	Negative
244	D00211	D00729	Negative
245	D00211	D02483	Negative
246	D00211	D03799	Negative
247	D00211	D06645	Negative
248	D00217	D00222	Negative
249	D00217	D00236	Negative
250	D00217	D00397	Negative
251	D00217	D00455	Negative
252	D00217	D00743	Negative
253	D00217	D00970	Negative

254	D00217	D01402	Negative
255	D00217	D01708	Negative
256	D00217	D02060	Negative
257	D00217	D07132	Negative
258	D00217	D08249	Negative
259	D00222	D00267	Negative
260	D00222	D00397	Negative
261	D00222	D00443	Negative
262	D00222	D00455	Negative
263	D00222	D00523	Negative
264	D00222	D00551	Negative
265	D00222	D00623	Negative
266	D00222	D00653	Negative
267	D00222	D00676	Negative
268	D00222	D01340	Negative
269	D00222	D02227	Negative
270	D00222	D05277	Negative
271	D00235	D00402	Negative
272	D00235	D00615	Negative
273	D00235	D01294	Negative
274	D00235	D02076	Negative
275	D00235	D02082	Negative
276	D00236	D00346	Negative
277	D00236	D00634	Negative
278	D00236	D00653	Negative
279	D00246	D00383	Negative
280	D00246	D00516	Negative
281	D00246	D00604	Negative
282	D00246	D00738	Negative
283	D00246	D00842	Negative
284	D00246	D01450	Negative
285	D00246	D02194	Negative
286	D00246	D05209	Negative
287	D00251	D00282	Negative
288	D00251	D00554	Negative
289	D00251	D00620	Negative
290	D00251	D00626	Negative
291	D00251	D00679	Negative
292	D00251	D00887	Negative
293	D00251	D01302	Negative
294	D00267	D00302	Negative
295	D00267	D00455	Negative
296	D00267	D00528	Negative

297	D00267	D00690	Negative
298	D00267	D00729	Negative
299	D00267	D00945	Negative
300	D00267	D01002	Negative
301	D00267	D03048	Negative
302	D00267	D03820	Negative
303	D00272	D00459	Negative
304	D00272	D00558	Negative
305	D00272	D00627	Negative
306	D00272	D00847	Negative
307	D00282	D00847	Negative
308	D00282	D02082	Negative
309	D00282	D05209	Negative
310	D00282	D08996	Negative
311	D00292	D00443	Negative
312	D00292	D00593	Negative
313	D00292	D02095	Negative
314	D00292	D08205	Negative
315	D00302	D00335	Negative
316	D00302	D00359	Negative
317	D00302	D00455	Negative
318	D00302	D00953	Negative
319	D00302	D01373	Negative
320	D00302	D02367	Negative
321	D00335	D00469	Negative
322	D00335	D00516	Negative
323	D00335	D00558	Negative
324	D00335	D02082	Negative
325	D00335	D02227	Negative
326	D00336	D00346	Negative
327	D00336	D00482	Negative
328	D00336	D00650	Negative
329	D00336	D00738	Negative
330	D00340	D00397	Negative
331	D00340	D01689	Negative
332	D00340	D07132	Negative
333	D00346	D00455	Negative
334	D00346	D00485	Negative
335	D00346	D00676	Negative
336	D00346	D02227	Negative
337	D00357	D00402	Negative
338	D00357	D00469	Negative
339	D00357	D00558	Negative

340	D00357	D00570	Negative
341	D00357	D01373	Negative
342	D00357	D02101	Negative
343	D00357	D02272	Negative
344	D00358	D00604	Negative
345	D00358	D00634	Negative
346	D00358	D00683	Negative
347	D00358	D00729	Negative
348	D00358	D01066	Negative
349	D00359	D00432	Negative
350	D00359	D00485	Negative
351	D00359	D00981	Negative
352	D00359	D01984	Negative
353	D00359	D02082	Negative
354	D00359	D07992	Negative
355	D00362	D00419	Negative
356	D00362	D00842	Negative
357	D00362	D00904	Negative
358	D00362	D00970	Negative
359	D00362	D01355	Negative
360	D00362	D02149	Negative
361	D00362	D03917	Negative
362	D00362	D06412	Negative
363	D00383	D00653	Negative
364	D00383	D00687	Negative
365	D00383	D00939	Negative
366	D00383	D00981	Negative
367	D00383	D02076	Negative
368	D00383	D04104	Negative
369	D00383	D08127	Negative
370	D00386	D00950	Negative
371	D00386	D00980	Negative
372	D00386	D00994	Negative
373	D00386	D02069	Negative
374	D00386	D08996	Negative
375	D00397	D00475	Negative
376	D00397	D00511	Negative
377	D00397	D01294	Negative
378	D00400	D00443	Negative
379	D00400	D00483	Negative
380	D00400	D00485	Negative
381	D00400	D00553	Negative
382	D00400	D00676	Negative

383	D00400	D02101	Negative
384	D00400	D03820	Negative
385	D00402	D00469	Negative
386	D00402	D00570	Negative
387	D00402	D00972	Negative
388	D00402	D02076	Negative
389	D00402	D03182	Negative
390	D00405	D00482	Negative
391	D00405	D00554	Negative
392	D00405	D01637	Negative
393	D00419	D00482	Negative
394	D00419	D00483	Negative
395	D00419	D00634	Negative
396	D00419	D01002	Negative
397	D00419	D01112	Negative
398	D00419	D05277	Negative
399	D00432	D00570	Negative
400	D00432	D00687	Negative
401	D00432	D00904	Negative
402	D00432	D01413	Negative
403	D00432	D01689	Negative
404	D00434	D00516	Negative
405	D00434	D00626	Negative
406	D00434	D00627	Negative
407	D00434	D00994	Negative
408	D00434	D02212	Negative
409	D00434	D06645	Negative
410	D00443	D00469	Negative
411	D00443	D00575	Negative
412	D00443	D04104	Negative
413	D00454	D00551	Negative
414	D00454	D00738	Negative
415	D00454	D00887	Negative
416	D00454	D00950	Negative
417	D00454	D02060	Negative
418	D00455	D00626	Negative
419	D00455	D00944	Negative
420	D00455	D01002	Negative
421	D00455	D01112	Negative
422	D00455	D02194	Negative
423	D00455	D06236	Negative
424	D00459	D00551	Negative
425	D00459	D00738	Negative

426	D00459	D01453	Negative
427	D00459	D02227	Negative
428	D00459	D02388	Negative
429	D00469	D00623	Negative
430	D00469	D00887	Negative
431	D00469	D01002	Negative
432	D00469	D01355	Negative
433	D00469	D02060	Negative
434	D00469	D02367	Negative
435	D00475	D00649	Negative
436	D00475	D00939	Negative
437	D00475	D01002	Negative
438	D00475	D02388	Negative
439	D00475	D03820	Negative
440	D00475	D03917	Negative
441	D00475	D05632	Negative
442	D00480	D00528	Negative
443	D00480	D00593	Negative
444	D00480	D00604	Negative
445	D00480	D01966	Negative
446	D00480	D02212	Negative
447	D00480	D05632	Negative
448	D00480	D07132	Negative
449	D00482	D00980	Negative
450	D00482	D01066	Negative
451	D00482	D02082	Negative
452	D00482	D02227	Negative
453	D00482	D07132	Negative
454	D00483	D00570	Negative
455	D00483	D00615	Negative
456	D00483	D00650	Negative
457	D00483	D00887	Negative
458	D00483	D02082	Negative
459	D00483	D02388	Negative
460	D00483	D03820	Negative
461	D00485	D00594	Negative
462	D00485	D00653	Negative
463	D00485	D02101	Negative
464	D00503	D00570	Negative
465	D00503	D01294	Negative
466	D00503	D01689	Negative
467	D00503	D05649	Negative
468	D00511	D00575	Negative

469	D00511	D00980	Negative
470	D00511	D01984	Negative
471	D00516	D00523	Negative
472	D00516	D00980	Negative
473	D00516	D03048	Negative
474	D00516	D06236	Negative
475	D00523	D00809	Negative
476	D00523	D00823	Negative
477	D00523	D00939	Negative
478	D00523	D00970	Negative
479	D00523	D01302	Negative
480	D00523	D02388	Negative
481	D00528	D00634	Negative
482	D00528	D00944	Negative
483	D00528	D00953	Negative
484	D00551	D00620	Negative
485	D00551	D00679	Negative
486	D00551	D02076	Negative
487	D00551	D08205	Negative
488	D00553	D00596	Negative
489	D00553	D00847	Negative
490	D00553	D00848	Negative
491	D00553	D01373	Negative
492	D00553	D02227	Negative
493	D00553	D05209	Negative
494	D00553	D08205	Negative
495	D00554	D00842	Negative
496	D00554	D02483	Negative
497	D00554	D07132	Negative
498	D00558	D01373	Negative
499	D00558	D01966	Negative
500	D00558	D05277	Negative
501	D00558	D07132	Negative
502	D00570	D00575	Negative
503	D00570	D00634	Negative
504	D00570	D00683	Negative
505	D00570	D00687	Negative
506	D00570	D00950	Negative
507	D00575	D02194	Negative
508	D00575	D08205	Negative
509	D00593	D00653	Negative
510	D00593	D00676	Negative
511	D00593	D01203	Negative

512	D00593	D01340	Negative
513	D00594	D00676	Negative
514	D00594	D00887	Negative
515	D00594	D00970	Negative
516	D00594	D01066	Negative
517	D00594	D01966	Negative
518	D00594	D02086	Negative
519	D00594	D06645	Negative
520	D00594	D08131	Negative
521	D00603	D00620	Negative
522	D00603	D00743	Negative
523	D00603	D08249	Negative
524	D00604	D01066	Negative
525	D00604	D01204	Negative
526	D00604	D02483	Negative
527	D00604	D03917	Negative
528	D00615	D00848	Negative
529	D00615	D01373	Negative
530	D00615	D01413	Negative
531	D00615	D01453	Negative
532	D00615	D01984	Negative
533	D00615	D02194	Negative
534	D00615	D03182	Negative
535	D00615	D03799	Negative
536	D00615	D07132	Negative
537	D00615	D08127	Negative
538	D00619	D00620	Negative
539	D00619	D00970	Negative
540	D00619	D02086	Negative
541	D00620	D01024	Negative
542	D00620	D05632	Negative
543	D00620	D06645	Negative
544	D00621	D00994	Negative
545	D00623	D00650	Negative
546	D00623	D00945	Negative
547	D00623	D01966	Negative
548	D00623	D02076	Negative
549	D00623	D02367	Negative
550	D00623	D03048	Negative
551	D00623	D08249	Negative
552	D00626	D02149	Negative
553	D00626	D03048	Negative
554	D00626	D03182	Negative

555	D00626	D03820	Negative
556	D00627	D00848	Negative
557	D00627	D00983	Negative
558	D00627	D01708	Negative
559	D00627	D07992	Negative
560	D00634	D00847	Negative
561	D00634	D01355	Negative
562	D00634	D02216	Negative
563	D00634	D03860	Negative
564	D00634	D05277	Negative
565	D00649	D01066	Negative
566	D00649	D02060	Negative
567	D00649	D03799	Negative
568	D00650	D00743	Negative
569	D00650	D01413	Negative
570	D00653	D00970	Negative
571	D00653	D01294	Negative
572	D00653	D05205	Negative
573	D00653	D06236	Negative
574	D00665	D00690	Negative
575	D00665	D00809	Negative
576	D00665	D01373	Negative
577	D00665	D02227	Negative
578	D00665	D02367	Negative
579	D00665	D02483	Negative
580	D00665	D08249	Negative
581	D00676	D00953	Negative
582	D00676	D00980	Negative
583	D00676	D01708	Negative
584	D00676	D02227	Negative
585	D00679	D02082	Negative
586	D00679	D02272	Negative
587	D00679	D06412	Negative
588	D00679	D06645	Negative
589	D00687	D00970	Negative
590	D00687	D01373	Negative
591	D00687	D02212	Negative
592	D00687	D03048	Negative
593	D00690	D00980	Negative
594	D00690	D01002	Negative
595	D00690	D01413	Negative
596	D00690	D03860	Negative
597	D00690	D03917	Negative

598	D00729	D01002	Negative
599	D00729	D02060	Negative
600	D00729	D02487	Negative
601	D00729	D06412	Negative
602	D00729	D08205	Negative
603	D00738	D00743	Negative
604	D00738	D00944	Negative
605	D00738	D08127	Negative
606	D00743	D00847	Negative
607	D00743	D00981	Negative
608	D00743	D02082	Negative
609	D00743	D02095	Negative
610	D00823	D00994	Negative
611	D00823	D02216	Negative
612	D00823	D02227	Negative
613	D00823	D03860	Negative
614	D00823	D04104	Negative
615	D00842	D01689	Negative
616	D00842	D02076	Negative
617	D00847	D00939	Negative
618	D00847	D00945	Negative
619	D00847	D02076	Negative
620	D00847	D02082	Negative
621	D00848	D00980	Negative
622	D00848	D00981	Negative
623	D00848	D01984	Negative
624	D00887	D00970	Negative
625	D00887	D02367	Negative
626	D00887	D05277	Negative
627	D00904	D00953	Negative
628	D00904	D02060	Negative
629	D00904	D05205	Negative
630	D00904	D08996	Negative
631	D00939	D00994	Negative
632	D00939	D01302	Negative
633	D00939	D01453	Negative
634	D00939	D01637	Negative
635	D00939	D02367	Negative
636	D00944	D01204	Negative
637	D00944	D02212	Negative
638	D00944	D03860	Negative
639	D00944	D05205	Negative
640	D00944	D07132	Negative

641	D00945	D00953	Negative
642	D00945	D00980	Negative
643	D00945	D01402	Negative
644	D00950	D00972	Negative
645	D00950	D01413	Negative
646	D00950	D05209	Negative
647	D00950	D08127	Negative
648	D00950	D08996	Negative
649	D00953	D00970	Negative
650	D00953	D01002	Negative
651	D00970	D01066	Negative
652	D00970	D02101	Negative
653	D00970	D08127	Negative
654	D00972	D01373	Negative
655	D00972	D02101	Negative
656	D00980	D00994	Negative
657	D00980	D01204	Negative
658	D00983	D01204	Negative
659	D00983	D01294	Negative
660	D00983	D01689	Negative
661	D00983	D02194	Negative
662	D00983	D07132	Negative
663	D00994	D01302	Negative
664	D01002	D01637	Negative
665	D01002	D02095	Negative
666	D01002	D08205	Negative
667	D01002	D08249	Negative
668	D01066	D01112	Negative
669	D01066	D01453	Negative
670	D01066	D07992	Negative
671	D01112	D01302	Negative
672	D01112	D01689	Negative
673	D01112	D02095	Negative
674	D01112	D06412	Negative
675	D01203	D01355	Negative
676	D01204	D01302	Negative
677	D01204	D02086	Negative
678	D01204	D02216	Negative
679	D01204	D03860	Negative
680	D01294	D01984	Negative
681	D01294	D02060	Negative
682	D01294	D06236	Negative
683	D01294	D07132	Negative

684	D01302	D01453	Negative
685	D01302	D05277	Negative
686	D01302	D06236	Negative
687	D01302	D08131	Negative
688	D01340	D08205	Negative
689	D01355	D01453	Negative
690	D01402	D02487	Negative
691	D01450	D05205	Negative
692	D01453	D02060	Negative
693	D01637	D02082	Negative
694	D01637	D02227	Negative
695	D01689	D02194	Negative
696	D01689	D02483	Negative
697	D01689	D05209	Negative
698	D01689	D06645	Negative
699	D01708	D07132	Negative
700	D01708	D07992	Negative
701	D01708	D08996	Negative
702	D01966	D05632	Negative
703	D01984	D03820	Negative
704	D02076	D02086	Negative
705	D02082	D03048	Negative
706	D02082	D05209	Negative
707	D02095	D03860	Negative
708	D02095	D05649	Negative
709	D02101	D06645	Negative
710	D02149	D06412	Negative
711	D02216	D02367	Negative
712	D02272	D04104	Negative
713	D02272	D05632	Negative
714	D02367	D02487	Negative
715	D02367	D06645	Negative
716	D02367	D08131	Negative
717	D02388	D03182	Negative
718	D02483	D08127	Negative
719	D02487	D03182	Negative
720	D03182	D03820	Negative
721	D03182	D05277	Negative
722	D03182	D05649	Negative
723	D03182	D08205	Negative
724	D05277	D07992	Negative
725	D06236	D07992	Negative
726	D06645	D08205	Negative
