# Supplementary material for: Prediction of Effective Drug Combinations by Chemical Interaction, Protein Interaction and Target Enrichment of KEGG Pathways
Source: Biomed Res Int. 2013 Sep 5;2013:723780. doi: 10.1155/2013/723780 (PMC3780555; doi:10.1155/2013/723780)
Supplement: Supplementary file 2 [file 723780.f2.pdf]

Supplementary Material II: Targets of 169 drugs investigated in the study.

Drug	Target
D00023	ENSP00000285379
D00023	ENSP00000344456
D00023	ENSP00000357066
D00049	ENSP00000299964
D00049	ENSP00000375066
D00049	ENSP00000378782
D00059	ENSP00000176183
D00059	ENSP00000306129
D00059	ENSP00000327652
D00059	ENSP00000354859
D00059	ENSP00000373169
D00068	ENSP00000023897
D00068	ENSP00000209668
D00068	ENSP00000218075
D00068	ENSP00000274576
D00068	ENSP00000306606
D00068	ENSP00000355155
D00088	ENSP00000231509
D00088	ENSP00000257497
D00088	ENSP00000327251
D00094	ENSP00000014914
D00094	ENSP00000237696
D00094	ENSP00000249750
D00094	ENSP00000254066
D00094	ENSP00000297785
D00094	ENSP00000352900
D00094	ENSP00000363812
D00094	ENSP00000368253
D00095	ENSP00000280155
D00095	ENSP00000303500
D00095	ENSP00000305372
D00095	ENSP00000306662
D00095	ENSP00000343782
D00095	ENSP00000358301
D00095	ENSP00000368766
D00095	ENSP00000369960
D00095	ENSP00000386069
D00095	ENSP00000387281
D00105	ENSP00000206249
D00105	ENSP00000336528

D00105	ENSP00000343925
D00109	ENSP00000287936
D00109	ENSP00000354612
D00109	ENSP00000356438
D00109	ENSP00000370254
D00113	ENSP00000255380
D00113	ENSP00000306490
D00113	ENSP00000319984
D00113	ENSP00000409378
D00118	ENSP00000354612
D00118	ENSP00000356438
D00126	ENSP00000354612
D00126	ENSP00000356438
D00145	ENSP00000315644
D00145	ENSP00000319170
D00145	ENSP00000396308
D00165	ENSP00000231509
D00165	ENSP00000257497
D00182	ENSP00000325120
D00186	ENSP00000396704
D00186	ENSP00000411532
D00188	ENSP00000229022
D00211	ENSP00000215591
D00211	ENSP00000228347
D00211	ENSP00000263331
D00211	ENSP00000263857
D00211	ENSP00000312735
D00211	ENSP00000314949
D00211	ENSP00000336528
D00211	ENSP00000361446
D00217	ENSP00000354612
D00217	ENSP00000356438
D00222	ENSP00000216367
D00222	ENSP00000217800
D00222	ENSP00000230859
D00222	ENSP00000231198
D00222	ENSP00000241436
D00222	ENSP00000242248
D00222	ENSP00000251607
D00222	ENSP00000264233
D00222	ENSP00000265421
D00222	ENSP00000268124
D00222	ENSP00000299206

D00222	ENSP00000322570
D00222	ENSP00000351697
D00222	ENSP00000361310
D00222	ENSP00000363284
D00222	ENSP00000368349
D00222	ENSP00000370968
D00222	ENSP00000420176
D00235	ENSP00000358301
D00236	ENSP00000219240
D00246	ENSP00000231509
D00251	ENSP00000219070
D00251	ENSP00000290866
D00251	ENSP00000361405
D00267	ENSP00000023897
D00267	ENSP00000264318
D00267	ENSP00000265294
D00267	ENSP00000274545
D00267	ENSP00000274547
D00267	ENSP00000295452
D00267	ENSP00000295454
D00267	ENSP00000299267
D00267	ENSP00000331912
D00267	ENSP00000335592
D00267	ENSP00000348897
D00267	ENSP00000359329
D00267	ENSP00000359334
D00267	ENSP00000359353
D00267	ENSP00000367848
D00267	ENSP00000386029
D00267	ENSP00000410732
D00267	ENSP00000412673
D00272	ENSP00000262502
D00272	ENSP00000370381
D00282	ENSP00000003100
D00282	ENSP00000262888
D00282	ENSP00000337915
D00292	ENSP00000231509
D00292	ENSP00000257497
D00292	ENSP00000327251
D00292	ENSP00000368253
D00302	ENSP00000270474
D00302	ENSP00000282096
D00302	ENSP00000341187

D00302	ENSP00000347046
D00302	ENSP00000351957
D00302	ENSP00000361850
D00302	ENSP00000361965
D00335	ENSP00000261200
D00335	ENSP00000287820
D00335	ENSP00000357068
D00335	ENSP00000374467
D00336	ENSP00000003084
D00336	ENSP00000261200
D00336	ENSP00000263817
D00336	ENSP00000339960
D00336	ENSP00000345708
D00336	ENSP00000363868
D00336	ENSP00000374467
D00340	ENSP00000178638
D00340	ENSP00000256119
D00340	ENSP00000262502
D00340	ENSP00000285379
D00340	ENSP00000286627
D00340	ENSP00000300900
D00340	ENSP00000367608
D00346	ENSP00000340684
D00346	ENSP00000367309
D00357	ENSP00000273430
D00358	ENSP00000275493
D00358	ENSP00000283254
D00358	ENSP00000283256
D00358	ENSP00000328968
D00358	ENSP00000346534
D00358	ENSP00000364554
D00358	ENSP00000386306
D00358	ENSP00000390600
D00358	ENSP00000396320
D00359	ENSP00000287936
D00359	ENSP00000349252
D00359	ENSP00000381331
D00362	ENSP00000252519
D00362	ENSP00000290866
D00383	ENSP00000290866
D00386	ENSP00000228916
D00386	ENSP00000300061
D00386	ENSP00000321594

D00386	ENSP00000345751
D00397	ENSP00000255380
D00397	ENSP00000306490
D00397	ENSP00000319984
D00397	ENSP00000409378
D00400	ENSP00000273430
D00402	ENSP00000256119
D00405	ENSP00000280155
D00405	ENSP00000350616
D00405	ENSP00000386069
D00405	ENSP00000387281
D00419	ENSP00000245457
D00419	ENSP00000291294
D00419	ENSP00000302846
D00419	ENSP00000349003
D00432	ENSP00000305372
D00432	ENSP00000343782
D00432	ENSP00000358301
D00434	ENSP00000287936
D00434	ENSP00000303242
D00443	ENSP00000350815
D00443	ENSP00000363822
D00454	ENSP00000176183
D00454	ENSP00000255380
D00454	ENSP00000276198
D00454	ENSP00000280155
D00454	ENSP00000289753
D00454	ENSP00000306129
D00454	ENSP00000306490
D00454	ENSP00000306662
D00454	ENSP00000307766
D00454	ENSP00000313661
D00454	ENSP00000316244
D00454	ENSP00000319984
D00454	ENSP00000327652
D00454	ENSP00000337949
D00454	ENSP00000347754
D00454	ENSP00000354859
D00454	ENSP00000358963
D00454	ENSP00000367959
D00454	ENSP00000368766
D00454	ENSP00000369960
D00454	ENSP00000373169

D00454	ENSP00000386069
D00454	ENSP00000387281
D00454	ENSP00000409378
D00455	ENSP00000262623
D00455	ENSP00000334216
D00459	ENSP00000290866
D00469	ENSP00000264381
D00469	ENSP00000303211
D00475	ENSP00000227638
D00475	ENSP00000301891
D00475	ENSP00000367102
D00480	ENSP00000255380
D00480	ENSP00000272298
D00480	ENSP00000291295
D00480	ENSP00000306490
D00480	ENSP00000319984
D00480	ENSP00000349467
D00480	ENSP00000354859
D00480	ENSP00000367959
D00480	ENSP00000369960
D00480	ENSP00000409378
D00482	ENSP00000234961
D00482	ENSP00000265572
D00482	ENSP00000394624
D00483	ENSP00000305372
D00483	ENSP00000316244
D00483	ENSP00000343782
D00483	ENSP00000358301
D00483	ENSP00000358963
D00485	ENSP00000261707
D00485	ENSP00000270349
D00485	ENSP00000280155
D00485	ENSP00000305372
D00485	ENSP00000306662
D00485	ENSP00000343782
D00485	ENSP00000358301
D00485	ENSP00000368766
D00485	ENSP00000369237
D00485	ENSP00000369960
D00485	ENSP00000386069
D00485	ENSP00000387281
D00503	ENSP00000272298
D00503	ENSP00000291295

D00503	ENSP00000327652
D00503	ENSP00000349467
D00503	ENSP00000354859
D00511	ENSP00000306662
D00511	ENSP00000368766
D00511	ENSP00000369960
D00516	ENSP00000357669
D00523	ENSP00000273430
D00523	ENSP00000360266
D00528	ENSP00000304501
D00528	ENSP00000308549
D00528	ENSP00000332116
D00528	ENSP00000336630
D00528	ENSP00000352608
D00551	ENSP00000283254
D00551	ENSP00000283256
D00551	ENSP00000328968
D00551	ENSP00000346534
D00551	ENSP00000364554
D00551	ENSP00000386306
D00551	ENSP00000390600
D00551	ENSP00000396320
D00553	ENSP00000283254
D00553	ENSP00000283256
D00553	ENSP00000328968
D00553	ENSP00000364554
D00553	ENSP00000385019
D00553	ENSP00000386796
D00553	ENSP00000390600
D00554	ENSP00000206249
D00554	ENSP00000336528
D00554	ENSP00000343925
D00558	ENSP00000350616
D00570	ENSP00000217133
D00570	ENSP00000259818
D00570	ENSP00000264071
D00570	ENSP00000309431
D00570	ENSP00000318697
D00570	ENSP00000320295
D00570	ENSP00000328808
D00570	ENSP00000341289
D00570	ENSP00000369703
D00570	ENSP00000399155

D00570	ENSP00000400663
D00570	ENSP00000401317
D00570	ENSP00000410071
D00570	ENSP00000410829
D00575	ENSP00000206249
D00593	ENSP00000261200
D00593	ENSP00000345708
D00593	ENSP00000374467
D00594	ENSP00000261200
D00594	ENSP00000287820
D00594	ENSP00000374467
D00596	ENSP00000287820
D00596	ENSP00000339787
D00603	ENSP00000305372
D00603	ENSP00000343782
D00603	ENSP00000358301
D00604	ENSP00000280155
D00604	ENSP00000386069
D00604	ENSP00000387281
D00615	ENSP00000256119
D00615	ENSP00000266376
D00615	ENSP00000288139
D00615	ENSP00000288197
D00615	ENSP00000320025
D00615	ENSP00000349320
D00615	ENSP00000355192
D00615	ENSP00000365441
D00615	ENSP00000377840
D00619	ENSP00000221444
D00619	ENSP00000261707
D00619	ENSP00000262186
D00619	ENSP00000266376
D00619	ENSP00000288139
D00619	ENSP00000301050
D00619	ENSP00000320025
D00619	ENSP00000328968
D00619	ENSP00000345708
D00619	ENSP00000352011
D00619	ENSP00000353362
D00619	ENSP00000355192
D00619	ENSP00000358784
D00619	ENSP00000358786
D00619	ENSP00000365441

D00619	ENSP00000376966
D00619	ENSP00000377840
D00619	ENSP00000383330
D00619	ENSP00000385019
D00620	ENSP00000290866
D00621	ENSP00000290866
D00623	ENSP00000252519
D00623	ENSP00000290866
D00626	ENSP00000230882
D00626	ENSP00000269571
D00626	ENSP00000273430
D00626	ENSP00000360973
D00627	ENSP00000273430
D00627	ENSP00000287820
D00634	ENSP00000305372
D00634	ENSP00000358301
D00649	ENSP00000225823
D00649	ENSP00000228468
D00649	ENSP00000228916
D00649	ENSP00000263980
D00649	ENSP00000300061
D00649	ENSP00000321594
D00649	ENSP00000345751
D00649	ENSP00000354193
D00649	ENSP00000361850
D00650	ENSP00000256119
D00650	ENSP00000262502
D00650	ENSP00000285379
D00650	ENSP00000286627
D00650	ENSP00000300900
D00650	ENSP00000370381
D00653	ENSP00000178638
D00653	ENSP00000256119
D00653	ENSP00000285379
D00653	ENSP00000285381
D00653	ENSP00000300900
D00653	ENSP00000309649
D00653	ENSP00000314099
D00653	ENSP00000314407
D00653	ENSP00000318912
D00653	ENSP00000345659
D00653	ENSP00000358107
D00653	ENSP00000366662

D00653	ENSP00000367608
D00665	ENSP00000261707
D00665	ENSP00000270349
D00665	ENSP00000369237
D00676	ENSP00000313661
D00676	ENSP00000316244
D00676	ENSP00000322924
D00676	ENSP00000358963
D00679	ENSP00000280155
D00679	ENSP00000306662
D00679	ENSP00000313661
D00679	ENSP00000316244
D00679	ENSP00000354859
D00679	ENSP00000358963
D00679	ENSP00000367959
D00679	ENSP00000368766
D00679	ENSP00000369237
D00679	ENSP00000369960
D00679	ENSP00000387281
D00683	ENSP00000305372
D00687	ENSP00000305372
D00690	ENSP00000231509
D00729	ENSP00000234961
D00729	ENSP00000264708
D00729	ENSP00000265572
D00729	ENSP00000272298
D00729	ENSP00000291295
D00729	ENSP00000349467
D00729	ENSP00000353362
D00729	ENSP00000394624
D00738	ENSP00000283254
D00738	ENSP00000283256
D00738	ENSP00000328968
D00738	ENSP00000346534
D00738	ENSP00000364554
D00738	ENSP00000386306
D00738	ENSP00000390600
D00738	ENSP00000396320
D00743	ENSP00000306662
D00743	ENSP00000368766
D00743	ENSP00000369960
D00809	ENSP00000228858
D00809	ENSP00000234961

D00809	ENSP00000255380
D00809	ENSP00000261707
D00809	ENSP00000265572
D00809	ENSP00000277120
D00809	ENSP00000280155
D00809	ENSP00000306490
D00809	ENSP00000316244
D00809	ENSP00000319591
D00809	ENSP00000319984
D00809	ENSP00000333496
D00809	ENSP00000352035
D00809	ENSP00000367959
D00809	ENSP00000368766
D00809	ENSP00000369237
D00809	ENSP00000369960
D00809	ENSP00000409378
D00823	ENSP00000261707
D00823	ENSP00000367959
D00842	ENSP00000234961
D00842	ENSP00000265572
D00842	ENSP00000394624
D00847	ENSP00000234961
D00847	ENSP00000265572
D00847	ENSP00000394624
D00848	ENSP00000261707
D00848	ENSP00000277010
D00848	ENSP00000279593
D00848	ENSP00000332549
D00848	ENSP00000355155
D00848	ENSP00000360616
D00887	ENSP00000242057
D00887	ENSP00000287936
D00887	ENSP00000353731
D00904	ENSP00000228468
D00904	ENSP00000352035
D00904	ENSP00000354612
D00904	ENSP00000356438
D00904	ENSP00000363512
D00904	ENSP00000364252
D00904	ENSP00000373648
D00904	ENSP00000396320
D00939	ENSP00000254667
D00939	ENSP00000263708

D00939	ENSP00000273398
D00939	ENSP00000349078
D00939	ENSP00000349932
D00944	ENSP00000229328
D00944	ENSP00000346148
D00944	ENSP00000360290
D00945	ENSP00000287820
D00950	ENSP00000206249
D00950	ENSP00000274192
D00950	ENSP00000325120
D00950	ENSP00000363822
D00953	ENSP00000325120
D00970	ENSP00000354612
D00970	ENSP00000356438
D00972	ENSP00000231509
D00980	ENSP00000231509
D00981	ENSP00000231509
D00983	ENSP00000231509
D00994	ENSP00000264381
D00994	ENSP00000303211
D01002	ENSP00000255380
D01002	ENSP00000306490
D01002	ENSP00000319984
D01002	ENSP00000409378
D01024	ENSP00000306662
D01024	ENSP00000368766
D01024	ENSP00000369960
D01066	ENSP00000234179
D01066	ENSP00000263125
D01066	ENSP00000263431
D01066	ENSP00000291281
D01066	ENSP00000291906
D01066	ENSP00000295797
D01066	ENSP00000305355
D01066	ENSP00000306124
D01066	ENSP00000329127
D01066	ENSP00000331602
D01066	ENSP00000333568
D01066	ENSP00000343325
D01066	ENSP00000359552
D01066	ENSP00000367830
D01112	ENSP00000254066
D01112	ENSP00000332296

D01112	ENSP00000352900
D01112	ENSP00000363812
D01112	ENSP00000419692
D01203	ENSP00000319591
D01203	ENSP00000334198
D01204	ENSP00000273430
D01294	ENSP00000206249
D01294	ENSP00000325120
D01302	ENSP00000263556
D01302	ENSP00000312326
D01340	ENSP00000206249
D01340	ENSP00000234961
D01340	ENSP00000265572
D01340	ENSP00000387699
D01340	ENSP00000394624
D01355	ENSP00000234961
D01355	ENSP00000255380
D01355	ENSP00000261707
D01355	ENSP00000265572
D01355	ENSP00000276198
D01355	ENSP00000299847
D01355	ENSP00000303727
D01355	ENSP00000355155
D01355	ENSP00000369237
D01355	ENSP00000394624
D01373	ENSP00000305372
D01402	ENSP00000231509
D01413	ENSP00000206249
D01413	ENSP00000336528
D01413	ENSP00000343925
D01450	ENSP00000283254
D01450	ENSP00000283256
D01450	ENSP00000292513
D01450	ENSP00000328968
D01450	ENSP00000346534
D01450	ENSP00000364554
D01450	ENSP00000386306
D01450	ENSP00000390600
D01450	ENSP00000396320
D01453	ENSP00000304501
D01453	ENSP00000308549
D01453	ENSP00000332116
D01453	ENSP00000336630

D01453	ENSP00000352608
D01637	ENSP00000231509
D01689	ENSP00000231509
D01708	ENSP00000231509
D01708	ENSP00000325120
D01708	ENSP00000350815
D01708	ENSP00000356436
D01966	ENSP00000289547
D01966	ENSP00000300060
D01966	ENSP00000356591
D01984	ENSP00000262623
D01984	ENSP00000334216
D02060	ENSP00000262461
D02060	ENSP00000264930
D02060	ENSP00000318557
D02060	ENSP00000346112
D02060	ENSP00000370381
D02060	ENSP00000387694
D02069	ENSP00000255380
D02069	ENSP00000306490
D02069	ENSP00000319984
D02069	ENSP00000409378
D02076	ENSP00000280155
D02076	ENSP00000386069
D02076	ENSP00000387281
D02082	ENSP00000273430
D02086	ENSP00000275493
D02086	ENSP00000283254
D02086	ENSP00000283256
D02086	ENSP00000328968
D02086	ENSP00000346534
D02086	ENSP00000364554
D02086	ENSP00000386306
D02086	ENSP00000390600
D02086	ENSP00000396320
D02095	ENSP00000234961
D02095	ENSP00000265572
D02095	ENSP00000277010
D02095	ENSP00000394624
D02101	ENSP00000234961
D02101	ENSP00000265572
D02101	ENSP00000394624
D02149	ENSP00000280155

D02149	ENSP00000303500
D02149	ENSP00000305372
D02149	ENSP00000306662
D02149	ENSP00000343782
D02149	ENSP00000358301
D02149	ENSP00000368766
D02149	ENSP00000369960
D02149	ENSP00000386069
D02149	ENSP00000387281
D02194	ENSP00000261615
D02212	ENSP00000255380
D02212	ENSP00000306490
D02212	ENSP00000319984
D02212	ENSP00000409378
D02216	ENSP00000396704
D02216	ENSP00000411532
D02227	ENSP00000265572
D02227	ENSP00000277010
D02227	ENSP00000394624
D02272	ENSP00000262186
D02272	ENSP00000263372
D02272	ENSP00000283254
D02272	ENSP00000283256
D02272	ENSP00000328968
D02272	ENSP00000346534
D02272	ENSP00000355580
D02272	ENSP00000364554
D02272	ENSP00000386306
D02272	ENSP00000396320
D02367	ENSP00000206249
D02367	ENSP00000325120
D02388	ENSP00000280155
D02388	ENSP00000386069
D02388	ENSP00000387281
D02483	ENSP00000261173
D02483	ENSP00000262429
D02483	ENSP00000263519
D02483	ENSP00000324172
D02483	ENSP00000324892
D02483	ENSP00000349595
D02483	ENSP00000350310
D02483	ENSP00000352665
D02483	ENSP00000353072

D02487	ENSP00000281243
D02487	ENSP00000319170
D02487	ENSP00000396308
D03048	ENSP00000265986
D03048	ENSP00000323929
D03182	ENSP00000023897
D03182	ENSP00000264318
D03182	ENSP00000264426
D03182	ENSP00000274545
D03182	ENSP00000303727
D03182	ENSP00000335592
D03182	ENSP00000348897
D03182	ENSP00000359285
D03182	ENSP00000359334
D03182	ENSP00000397026
D03799	ENSP00000325120
D03820	ENSP00000274192
D03860	ENSP00000234961
D03860	ENSP00000394624
D03917	ENSP00000325120
D03917	ENSP00000350815
D03917	ENSP00000363822
D04104	ENSP00000206249
D04104	ENSP00000325120
D05205	ENSP00000206249
D05205	ENSP00000325120
D05209	ENSP00000206249
D05209	ENSP00000325120
D05277	ENSP00000305372
D05632	ENSP00000394624
D05649	ENSP00000261707
D05649	ENSP00000270349
D05649	ENSP00000280155
D05649	ENSP00000305372
D05649	ENSP00000306662
D05649	ENSP00000343782
D05649	ENSP00000358301
D05649	ENSP00000368766
D05649	ENSP00000369237
D05649	ENSP00000369960
D05649	ENSP00000386069
D05649	ENSP00000387281
D06236	ENSP00000315644

D06236	ENSP00000319170
D06236	ENSP00000396308
D06412	ENSP00000272190
D06645	ENSP00000353731
D07132	ENSP00000234961
D07132	ENSP00000265572
D07132	ENSP00000394624
D07992	ENSP00000290866
D08127	ENSP00000275493
D08127	ENSP00000283254
D08127	ENSP00000283256
D08127	ENSP00000328968
D08127	ENSP00000346534
D08127	ENSP00000364554
D08127	ENSP00000386306
D08127	ENSP00000390600
D08127	ENSP00000396320
D08131	ENSP00000252519
D08131	ENSP00000290866
D08205	ENSP00000280155
D08205	ENSP00000350616
D08205	ENSP00000386069
D08205	ENSP00000387281
D08249	ENSP00000206249
D08249	ENSP00000234961
D08249	ENSP00000265572
D08249	ENSP00000387699
D08249	ENSP00000394624
D08996	ENSP00000353731
