# Supplementary material for: Prediction of Effective Drug Combinations by Chemical Interaction, Protein Interaction and Target Enrichment of KEGG Pathways
Source: Biomed Res Int. 2013 Sep 5;2013:723780. doi: 10.1155/2013/723780 (PMC3780555; doi:10.1155/2013/723780)
Supplement: Supplementary file 3 [file 723780.f3.pdf]

Supplementary Material III: Two lists obtained by mRMR

Note: the one-to-one correspondence of pathway ID and pathway name can be found in the third table in this file.

(I) MaxRel features list

Order	Feature name
1	F1[a]
2	hsa04964_1+hsa04964_2[h]
3	F2[b]
4	hsa00052_1+hsa00052_2
5	hsa04970_1+hsa04970_2
6	abs(hsa00910_1-hsa00910_2)[i]
7	hsa00910_1+hsa00910_2
8	abs(hsa05215_1-hsa05215_2)
9	abs(hsa05130_1-hsa05130_2)
10	abs(hsa00520_1-hsa00520_2)
11	hsa05211_1+hsa05211_2
12	hsa04960_1+hsa04960_2
13	hsa05130_1+hsa05130_2
14	abs(hsa04973_1-hsa04973_2)
15	hsa04972_1+hsa04972_2
16	hsa00520_1+hsa00520_2
17	hsa05110_1+hsa05110_2
18	abs(hsa04964_1-hsa04964_2)
19	abs(hsa04115_1-hsa04115_2)
20	F3[c]
21	abs(hsa04340_1-hsa04340_2)
22	hsa05100_1+hsa05100_2
23	hsa05214_1+hsa05214_2
24	abs(hsa04320_1-hsa04320_2)
25	hsa05020_1+hsa05020_2
26	hsa04330_1+hsa04330_2
27	abs(hsa05110_1-hsa05110_2)
28	abs(hsa04970_1-hsa04970_2)
29	hsa04145_1+hsa04145_2
30	hsa04150_1+hsa04150_2
31	hsa04622_1+hsa04622_2
32	hsa04620_1+hsa04620_2
33	abs(hsa05222_1-hsa05222_2)
34	abs(hsa05220_1-hsa05220_2)
35	hsa04910_1+hsa04910_2
36	hsa04930_1+hsa04930_2

37	abs(hsa04330_1-hsa04330_2)
38	abs(hsa05150_1-hsa05150_2)
39	hsa04210_1+hsa04210_2
40	hsa04973_1+hsa04973_2
41	hsa05218_1+hsa05218_2
42	hsa05213_1+hsa05213_2
43	hsa04621_1+hsa04621_2
44	hsa05222_1+hsa05222_2
45	abs(hsa05217_1-hsa05217_2)
46	hsa05143_1+hsa05143_2
47	abs(hsa04720_1-hsa04720_2)
48	hsa05215_1+hsa05215_2
49	hsa04115_1+hsa04115_2
50	hsa04380_1+hsa04380_2
51	hsa04722_1+hsa04722_2
52	abs(hsa00140_1-hsa00140_2)
53	hsa05010_1+hsa05010_2
54	abs(hsa05131_1-hsa05131_2)
55	hsa04310_1+hsa04310_2
56	abs(hsa00830_1-hsa00830_2)
57	hsa05142_1+hsa05142_2
58	hsa05221_1+hsa05221_2
59	abs(hsa05218_1-hsa05218_2)
60	hsa00430_1+hsa00430_2
61	abs(hsa04662_1-hsa04662_2)
62	abs(hsa00604_1-hsa00604_2)
63	hsa05120_1+hsa05120_2
64	hsa04662_1+hsa04662_2
65	abs(hsa04120_1-hsa04120_2)
66	abs(hsa00052_1-hsa00052_2)
67	abs(hsa04370_1-hsa04370_2)
68	hsa04666_1+hsa04666_2
69	abs(hsa04360_1-hsa04360_2)
70	hsa05160_1+hsa05160_2
71	hsa04114_1+hsa04114_2
72	abs(hsa04070_1-hsa04070_2)
73	hsa05216_1+hsa05216_2
74	abs(hsa04310_1-hsa04310_2)
75	abs(hsa00430_1-hsa00430_2)
76	hsa00480_1+hsa00480_2
77	hsa04660_1+hsa04660_2
78	abs(hsa05146_1-hsa05146_2)
79	hsa05145_1+hsa05145_2

80	abs(hsa04540_1-hsa04540_2)
81	abs(hsa04010_1-hsa04010_2)
82	abs(hsa04960_1-hsa04960_2)
83	hsa05416_1+hsa05416_2
84	abs(hsa05140_1-hsa05140_2)
85	abs(hsa03450_1-hsa03450_2)
86	hsa04720_1+hsa04720_2
87	abs(hsa00480_1-hsa00480_2)
88	abs(hsa04650_1-hsa04650_2)
89	hsa05014_1+hsa05014_2
90	abs(hsa04660_1-hsa04660_2)
91	hsa04971_1+hsa04971_2
92	hsa04530_1+hsa04530_2
93	abs(hsa05020_1-hsa05020_2)
94	hsa00130_1+hsa00130_2
95	hsa05212_1+hsa05212_2
96	abs(hsa04145_1-hsa04145_2)
97	hsa04920_1+hsa04920_2
98	abs(hsa04664_1-hsa04664_2)
99	hsa00920_1+hsa00920_2
100	hsa04010_1+hsa04010_2
101	abs(hsa05010_1-hsa05010_2)
102	hsa00982_1+hsa00982_2
103	abs(hsa04614_1-hsa04614_2)
104	hsa04350_1+hsa04350_2
105	abs(hsa04621_1-hsa04621_2)
106	abs(hsa00232_1-hsa00232_2)
107	abs(hsa00564_1-hsa00564_2)
108	abs(hsa05143_1-hsa05143_2)
109	abs(hsa04623_1-hsa04623_2)
110	abs(hsa05214_1-hsa05214_2)
111	abs(hsa00591_1-hsa00591_2)
112	abs(hsa04912_1-hsa04912_2)
113	abs(hsa04914_1-hsa04914_2)
114	hsa00511_1+hsa00511_2
115	abs(hsa03410_1-hsa03410_2)
116	hsa04520_1+hsa04520_2
117	abs(hsa04976_1-hsa04976_2)
118	abs(hsa03420_1-hsa03420_2)
119	abs(hsa04975_1-hsa04975_2)
120	hsa04012_1+hsa04012_2
121	abs(hsa04620_1-hsa04620_2)
122	abs(hsa05200_1-hsa05200_2)

123	abs(hsa00590_1-hsa00590_2)
124	hsa05131_1+hsa05131_2
125	abs(hsa00592_1-hsa00592_2)
126	abs(hsa00270_1-hsa00270_2)
127	hsa05200_1+hsa05200_2
128	abs(hsa00785_1-hsa00785_2)
129	hsa05016_1+hsa05016_2
130	abs(hsa04972_1-hsa04972_2)
131	abs(hsa04977_1-hsa04977_2)
132	abs(hsa05145_1-hsa05145_2)
133	hsa04120_1+hsa04120_2
134	abs(hsa04110_1-hsa04110_2)
135	hsa00531_1+hsa00531_2
136	hsa04623_1+hsa04623_2
137	hsa00030_1+hsa00030_2
138	abs(hsa00562_1-hsa00562_2)
139	hsa05220_1+hsa05220_2
140	abs(hsa00240_1-hsa00240_2)
141	hsa05217_1+hsa05217_2
142	abs(hsa00565_1-hsa00565_2)
143	abs(hsa03030_1-hsa03030_2)
144	abs(hsa04012_1-hsa04012_2)
145	hsa00020_1+hsa00020_2
146	abs(hsa00472_1-hsa00472_2)
147	abs(hsa03018_1-hsa03018_2)
148	abs(hsa01100_1-hsa01100_2)
149	abs(hsa00030_1-hsa00030_2)
150	hsa01100_1+hsa01100_2
151	hsa00562_1+hsa00562_2
152	hsa04370_1+hsa04370_2
153	abs(hsa05221_1-hsa05221_2)
154	hsa00564_1+hsa00564_2
155	hsa04975_1+hsa04975_2
156	abs(hsa03430_1-hsa03430_2)
157	abs(hsa04930_1-hsa04930_2)
158	hsa03410_1+hsa03410_2
159	hsa00565_1+hsa00565_2
160	hsa00590_1+hsa00590_2
161	abs(hsa05016_1-hsa05016_2)
162	abs(hsa00630_1-hsa00630_2)
163	hsa04940_1+hsa04940_2
164	abs(hsa05212_1-hsa05212_2)
165	abs(hsa00524_1-hsa00524_2)

166	hsa00532_1+hsa00532_2
167	abs(hsa00982_1-hsa00982_2)
168	hsa04110_1+hsa04110_2
169	abs(hsa05223_1-hsa05223_2)
170	hsa00592_1+hsa00592_2
171	hsa00120_1+hsa00120_2
172	hsa00380_1+hsa00380_2
173	hsa00010_1+hsa00010_2
174	hsa00591_1+hsa00591_2
175	hsa05223_1+hsa05223_2
176	hsa00785_1+hsa00785_2
177	hsa05210_1+hsa05210_2
178	hsa04540_1+hsa04540_2
179	hsa00061_1+hsa00061_2
180	abs(hsa00310_1-hsa00310_2)
181	abs(hsa04380_1-hsa04380_2)
182	abs(hsa00450_1-hsa00450_2)
183	abs(hsa00531_1-hsa00531_2)
184	abs(hsa00290_1-hsa00290_2)
185	hsa03430_1+hsa03430_2
186	abs(hsa04150_1-hsa04150_2)
187	hsa00604_1+hsa00604_2
188	abs(hsa04910_1-hsa04910_2)
189	hsa00360_1+hsa00360_2
190	abs(hsa03440_1-hsa03440_2)
191	hsa04977_1+hsa04977_2
192	hsa05146_1+hsa05146_2
193	abs(hsa04744_1-hsa04744_2)
194	abs(hsa03013_1-hsa03013_2)
195	abs(hsa05012_1-hsa05012_2)
196	abs(hsa03040_1-hsa03040_2)
197	hsa03030_1+hsa03030_2
198	hsa00260_1+hsa00260_2
199	hsa04730_1+hsa04730_2
200	hsa00232_1+hsa00232_2
201	hsa04510_1+hsa04510_2
202	abs(hsa00380_1-hsa00380_2)
203	abs(hsa00514_1-hsa00514_2)
204	hsa05219_1+hsa05219_2
205	hsa00350_1+hsa00350_2
206	hsa00740_1+hsa00740_2
207	abs(hsa05219_1-hsa05219_2)
208	abs(hsa04940_1-hsa04940_2)

209	abs(hsa04971_1-hsa04971_2)
210	hsa00471_1+hsa00471_2
211	hsa00230_1+hsa00230_2
212	abs(hsa00230_1-hsa00230_2)
213	abs(hsa04670_1-hsa04670_2)
214	abs(hsa04666_1-hsa04666_2)
215	abs(hsa03050_1-hsa03050_2)
216	abs()
217	hsa00563_1+hsa00563_2
218	abs(hsa00510_1-hsa00510_2)
219	hsa04664_1+hsa04664_2
220	hsa00330_1+hsa00330_2
221	abs(hsa04080_1-hsa04080_2)
222	abs(hsa00280_1-hsa00280_2)
223	hsa00270_1+hsa00270_2
224	F7[g]
225	hsa04742_1+hsa04742_2
226	hsa03440_1+hsa03440_2
227	hsa04650_1+hsa04650_2
228	abs(hsa00790_1-hsa00790_2)
229	abs(hsa05100_1-hsa05100_2)
230	hsa05320_1+hsa05320_2
231	hsa03015_1+hsa03015_2
232	abs(hsa04520_1-hsa04520_2)
233	abs(hsa05211_1-hsa05211_2)
234	hsa00340_1+hsa00340_2
235	abs(hsa00340_1-hsa00340_2)
236	hsa04142_1+hsa04142_2
237	abs(hsa00770_1-hsa00770_2)
238	abs(hsa05144_1-hsa05144_2)
239	hsa05012_1+hsa05012_2
240	F5[e]
241	hsa04130_1+hsa04130_2
242	hsa04672_1+hsa04672_2
243	abs(hsa04710_1-hsa04710_2)
244	abs(hsa00460_1-hsa00460_2)
245	abs(hsa00471_1-hsa00471_2)
246	abs(hsa00010_1-hsa00010_2)
247	abs(hsa04966_1-hsa04966_2)
248	abs(hsa03022_1-hsa03022_2)
249	abs(hsa04722_1-hsa04722_2)
250	hsa00290_1+hsa00290_2
251	hsa00053_1+hsa00053_2

252	abs(hsa04020_1-hsa04020_2)
253	hsa05414_1+hsa05414_2
254	abs(hsa00730_1-hsa00730_2)
255	hsa00730_1+hsa00730_2
256	abs(hsa04672_1-hsa04672_2)
257	abs(hsa00260_1-hsa00260_2)
258	abs(hsa00360_1-hsa00360_2)
259	abs(hsa00563_1-hsa00563_2)
260	abs(hsa00600_1-hsa00600_2)
261	abs(hsa05014_1-hsa05014_2)
262	abs(hsa00120_1-hsa00120_2)
263	abs(hsa04530_1-hsa04530_2)
264	hsa03013_1+hsa03013_2
265	hsa03040_1+hsa03040_2
266	hsa05310_1+hsa05310_2
267	hsa00240_1+hsa00240_2
268	abs(hsa05160_1-hsa05160_2)
269	abs(hsa04920_1-hsa04920_2)
270	abs(hsa00500_1-hsa00500_2)
271	abs(hsa05120_1-hsa05120_2)
272	hsa04070_1+hsa04070_2
273	hsa00860_1+hsa00860_2
274	abs(hsa05213_1-hsa05213_2)
275	hsa00603_1+hsa00603_2
276	abs(hsa00410_1-hsa00410_2)
277	hsa03020_1+hsa03020_2
278	abs(hsa03020_1-hsa03020_2)
279	abs(hsa00740_1-hsa00740_2)
280	hsa00450_1+hsa00450_2
281	abs(hsa04114_1-hsa04114_2)
282	hsa00460_1+hsa00460_2
283	hsa04966_1+hsa04966_2
284	hsa03008_1+hsa03008_2
285	abs(hsa03008_1-hsa03008_2)
286	hsa05150_1+hsa05150_2
287	abs(hsa04612_1-hsa04612_2)
288	abs(hsa05416_1-hsa05416_2)
289	hsa00760_1+hsa00760_2
290	abs(hsa05210_1-hsa05210_2)
291	abs(hsa00300_1-hsa00300_2)
292	hsa00190_1+hsa00190_2
293	abs(hsa00190_1-hsa00190_2)
294	abs(hsa03015_1-hsa03015_2)

295	abs(hsa04060_1-hsa04060_2)
296	hsa04976_1+hsa04976_2
297	hsa03050_1+hsa03050_2
298	hsa04916_1+hsa04916_2
299	hsa05144_1+hsa05144_2
300	hsa05140_1+hsa05140_2
301	abs(hsa04640_1-hsa04640_2)
302	abs(hsa00760_1-hsa00760_2)
303	hsa00514_1+hsa00514_2
304	hsa00040_1+hsa00040_2
305	hsa00983_1+hsa00983_2
306	abs(hsa05330_1-hsa05330_2)
307	hsa04360_1+hsa04360_2
308	hsa00510_1+hsa00510_2
309	abs(hsa05323_1-hsa05323_2)
310	hsa00670_1+hsa00670_2
311	abs(hsa00670_1-hsa00670_2)
312	hsa00780_1+hsa00780_2
313	abs(hsa00780_1-hsa00780_2)
314	abs(hsa04130_1-hsa04130_2)
315	abs(hsa00400_1-hsa00400_2)
316	abs(hsa00603_1-hsa00603_2)
317	hsa05330_1+hsa05330_2
318	hsa00980_1+hsa00980_2
319	hsa04914_1+hsa04914_2
320	hsa00300_1+hsa00300_2
321	abs(hsa00601_1-hsa00601_2)
322	hsa04122_1+hsa04122_2
323	abs(hsa04122_1-hsa04122_2)
324	hsa00400_1+hsa00400_2
325	hsa04640_1+hsa04640_2
326	hsa04144_1+hsa04144_2
327	hsa00410_1+hsa00410_2
328	abs(hsa04950_1-hsa04950_2)
329	abs(hsa04062_1-hsa04062_2)
330	hsa04062_1+hsa04062_2
331	abs(hsa00532_1-hsa00532_2)
332	hsa04020_1+hsa04020_2
333	abs(hsa04730_1-hsa04730_2)
334	abs(hsa00620_1-hsa00620_2)
335	hsa00140_1+hsa00140_2
336	hsa04912_1+hsa04912_2
337	hsa00790_1+hsa00790_2

338	abs(hsa00130_1-hsa00130_2)
339	abs(hsa04974_1-hsa04974_2)
340	abs(hsa04810_1-hsa04810_2)
341	abs(hsa04140_1-hsa04140_2)
342	hsa00472_1+hsa00472_2
343	abs(hsa04962_1-hsa04962_2)
344	abs(hsa04514_1-hsa04514_2)
345	abs(hsa04350_1-hsa04350_2)
346	hsa00601_1+hsa00601_2
347	hsa04270_1+hsa04270_2
348	hsa04610_1+hsa04610_2
349	hsa04060_1+hsa04060_2
350	hsa04514_1+hsa04514_2
351	abs(hsa00350_1-hsa00350_2)
352	hsa04670_1+hsa04670_2
353	hsa04962_1+hsa04962_2
354	abs(hsa00980_1-hsa00980_2)
355	abs(hsa04916_1-hsa04916_2)
356	hsa00970_1+hsa00970_2
357	abs(hsa04270_1-hsa04270_2)
358	abs(hsa05216_1-hsa05216_2)
359	abs(hsa00020_1-hsa00020_2)
360	hsa04320_1+hsa04320_2
361	hsa04810_1+hsa04810_2
362	hsa00524_1+hsa00524_2
363	hsa05332_1+hsa05332_2
364	abs(hsa05414_1-hsa05414_2)
365	hsa04950_1+hsa04950_2
366	abs(hsa04622_1-hsa04622_2)
367	hsa04512_1+hsa04512_2
368	abs(hsa00053_1-hsa00053_2)
369	abs(hsa04141_1-hsa04141_2)
370	abs(hsa00061_1-hsa00061_2)
371	abs(hsa00970_1-hsa00970_2)
372	abs(hsa04142_1-hsa04142_2)
373	hsa05323_1+hsa05323_2
374	hsa00600_1+hsa00600_2
375	hsa00650_1+hsa00650_2
376	abs(hsa04144_1-hsa04144_2)
377	hsa03060_1+hsa03060_2
378	abs(hsa04210_1-hsa04210_2)
379	abs(hsa00040_1-hsa00040_2)
380	abs(hsa00250_1-hsa00250_2)

381	hsa04630_1+hsa04630_2
382	abs(hsa00983_1-hsa00983_2)
383	hsa00250_1+hsa00250_2
384	hsa00280_1+hsa00280_2
385	abs(hsa04510_1-hsa04510_2)
386	hsa00640_1+hsa00640_2
387	hsa03420_1+hsa03420_2
388	abs(hsa00071_1-hsa00071_2)
389	abs(hsa03060_1-hsa03060_2)
390	hsa00534_1+hsa00534_2
391	abs(hsa04742_1-hsa04742_2)
392	abs(hsa02010_1-hsa02010_2)
393	abs(hsa00330_1-hsa00330_2)
394	hsa04612_1+hsa04612_2
395	hsa04974_1+hsa04974_2
396	abs(hsa05320_1-hsa05320_2)
397	hsa04744_1+hsa04744_2
398	hsa03018_1+hsa03018_2
399	hsa00830_1+hsa00830_2
400	hsa05340_1+hsa05340_2
401	abs(hsa03010_1-hsa03010_2)
402	hsa03010_1+hsa03010_2
403	hsa04140_1+hsa04140_2
404	abs(hsa05340_1-hsa05340_2)
405	abs(hsa00051_1-hsa00051_2)
406	abs(hsa00512_1-hsa00512_2)
407	abs(hsa00640_1-hsa00640_2)
408	hsa00072_1+hsa00072_2
409	abs(hsa05332_1-hsa05332_2)
410	abs(hsa00100_1-hsa00100_2)
411	hsa00100_1+hsa00100_2
412	hsa03320_1+hsa03320_2
413	abs(hsa00860_1-hsa00860_2)
414	hsa00310_1+hsa00310_2
415	hsa00620_1+hsa00620_2
416	abs(hsa04512_1-hsa04512_2)
417	abs(hsa00920_1-hsa00920_2)
418	hsa00900_1+hsa00900_2
419	abs(hsa00534_1-hsa00534_2)
420	hsa04740_1+hsa04740_2
421	abs(hsa04740_1-hsa04740_2)
422	hsa05410_1+hsa05410_2
423	hsa00062_1+hsa00062_2

424	hsa00561_1+hsa00561_2
425	hsa00071_1+hsa00071_2
426	abs(hsa04610_1-hsa04610_2)
427	hsa04710_1+hsa04710_2
428	hsa05412_1+hsa05412_2
429	hsa00500_1+hsa00500_2
430	hsa02010_1+hsa02010_2
431	hsa04340_1+hsa04340_2
432	hsa00750_1+hsa00750_2
433	abs(hsa00062_1-hsa00062_2)
434	abs(hsa00900_1-hsa00900_2)
435	abs(hsa05142_1-hsa05142_2)
436	hsa04141_1+hsa04141_2
437	hsa04080_1+hsa04080_2
438	abs(hsa00561_1-hsa00561_2)
439	abs(hsa00511_1-hsa00511_2)
440	abs(hsa03320_1-hsa03320_2)
441	hsa04260_1+hsa04260_2
442	hsa00630_1+hsa00630_2
443	abs(hsa05410_1-hsa05410_2)
444	hsa03022_1+hsa03022_2
445	hsa00533_1+hsa00533_2
446	abs(hsa04260_1-hsa04260_2)
447	F6[f]
448	abs(hsa05322_1-hsa05322_2)
449	hsa05322_1+hsa05322_2
450	hsa00051_1+hsa00051_2
451	hsa00770_1+hsa00770_2
452	abs(hsa04146_1-hsa04146_2)
453	hsa04146_1+hsa04146_2
454	abs(hsa05310_1-hsa05310_2)
455	hsa03450_1+hsa03450_2
456	abs(hsa00750_1-hsa00750_2)
457	abs(hsa05412_1-hsa05412_2)
458	abs(hsa00072_1-hsa00072_2)
459	hsa04614_1+hsa04614_2
460	hsa00512_1+hsa00512_2
461	abs(hsa00650_1-hsa00650_2)
462	abs(hsa04630_1-hsa04630_2)
463	hsa01040_1+hsa01040_2
464	abs(hsa00533_1-hsa00533_2)

[a] The confidence score of two drugs in the combination, see “Chemical interaction” of Section “Features of drug combinations” for detail.

[b] The maximum confidence score between targets of drug d_1 and targets of d_2, see **Eq. 1** and related text in Section “Features of drug combinations” for detail.

[c] The mean value of confidence scores between targets of drug d_1 and targets of d_2, see **Eq. 1** and related text in Section “Features of drug combinations” for detail.

[d] The absolute value of difference of maximum confidence score between targets of drug d_1 and maximum confidence score between targets of drug d_2, see **Eq. 2** and related text in Section “Features of drug combinations” for detail.

[e] The absolute value of difference of mean value of confidence scores between targets of drug d_1 and mean value of confidence score between targets of drug d_2, see **Eq. 2** and related text in Section “Features of drug combinations” for detail.

[f] The sum of maximum confidence score between targets of drug d_1 and maximum confidence score between targets of drug d_2, see **Eq. 2** and related text in Section “Features of drug combinations” for detail.

[g] The sum of mean value of confidence scores between targets of drug d_1 and mean value of confidence score between targets of drug d_2, see **Eq.2** and related text in Section “Features of drug combinations” for detail.

[h] The sum of enrichment score of drug d_1 and pathway hsa04964 (“Proximal tubule bicarbonate reclamation”) and enrichment score of drug d_2 and pathway hsa04964 (“Proximal tubule bicarbonate reclamation”), see **Eq. 3** and related text in Section “Features of drug combinations” for detail.

[i] The absolute value of the deference of enrichment score of drug d_1 and pathway hsa00910 (“Nitrogen metabolism”) and enrichment score of drug d_2 and pathway hsa00910 (“Nitrogen metabolism”), see **Eq. 3** and related text in Section “Features of drug combinations” for detail.

(II) mRMR features list

Order	Feature name
1	F1[a]
2	abs(hsa05215_1-hsa05215_2)[h]
3	hsa04964_1+hsa04964_2[i]
4	F2[b]
5	abs(hsa00140_1-hsa00140_2)
6	hsa04145_1+hsa04145_2
7	abs(hsa05150_1-hsa05150_2)
8	abs(hsa04973_1-hsa04973_2)
9	abs(hsa04340_1-hsa04340_2)
10	hsa00052_1+hsa00052_2
11	abs(hsa04310_1-hsa04310_2)
12	hsa00531_1+hsa00531_2
13	hsa04972_1+hsa04972_2
14	abs(hsa04976_1-hsa04976_2)
15	abs(hsa03018_1-hsa03018_2)
16	abs(hsa04744_1-hsa04744_2)
17	abs(hsa04977_1-hsa04977_2)
18	abs(hsa04330_1-hsa04330_2)
19	abs(hsa00430_1-hsa00430_2)
20	abs(hsa05130_1-hsa05130_2)
21	hsa00920_1+hsa00920_2
22	abs(hsa00785_1-hsa00785_2)
23	hsa05020_1+hsa05020_2
24	hsa00511_1+hsa00511_2
25	F3[c]
26	abs(hsa04320_1-hsa04320_2)
27	abs(hsa00520_1-hsa00520_2)
28	abs(hsa00310_1-hsa00310_2)
29	abs(hsa00270_1-hsa00270_2)
30	abs(hsa04115_1-hsa04115_2)
31	hsa04966_1+hsa04966_2
32	abs(hsa00830_1-hsa00830_2)
33	abs(hsa00910_1-hsa00910_2)
34	abs(hsa05217_1-hsa05217_2)
35	abs(hsa05010_1-hsa05010_2)
36	abs(hsa04150_1-hsa04150_2)
37	hsa00532_1+hsa00532_2
38	abs(hsa04514_1-hsa04514_2)
39	abs(hsa04975_1-hsa04975_2)
40	hsa05110_1+hsa05110_2

41	abs(hsa05146_1-hsa05146_2)
42	abs(hsa05012_1-hsa05012_2)
43	abs(hsa04614_1-hsa04614_2)
44	hsa04130_1+hsa04130_2
45	hsa00480_1+hsa00480_2
46	hsa05211_1+hsa05211_2
47	abs(hsa05322_1-hsa05322_2)
48	abs(hsa04964_1-hsa04964_2)
49	hsa04120_1+hsa04120_2
50	hsa00780_1+hsa00780_2
51	abs(hsa00630_1-hsa00630_2)
52	abs(hsa00510_1-hsa00510_2)
53	abs(hsa00061_1-hsa00061_2)
54	abs(hsa05020_1-hsa05020_2)
55	abs(hsa00232_1-hsa00232_2)
56	hsa00910_1+hsa00910_2
57	abs(hsa04070_1-hsa04070_2)
58	hsa04740_1+hsa04740_2
59	hsa04970_1+hsa04970_2
60	hsa00471_1+hsa00471_2
61	hsa04142_1+hsa04142_2
62	abs(hsa03010_1-hsa03010_2)
63	abs(hsa04080_1-hsa04080_2)
64	abs(hsa00300_1-hsa00300_2)
65	abs(hsa00400_1-hsa00400_2)
66	abs(hsa05120_1-hsa05120_2)
67	abs(hsa00760_1-hsa00760_2)
68	abs(hsa04960_1-hsa04960_2)
69	abs(hsa04950_1-hsa04950_2)
70	abs(hsa04540_1-hsa04540_2)
71	abs(hsa04670_1-hsa04670_2)
72	abs(hsa05110_1-hsa05110_2)
73	abs(hsa03450_1-hsa03450_2)
74	abs(hsa04940_1-hsa04940_2)
75	abs(hsa00472_1-hsa00472_2)
76	hsa03010_1+hsa03010_2
77	abs(hsa05016_1-hsa05016_2)
78	abs(hsa04910_1-hsa04910_2)
79	abs(hsa04370_1-hsa04370_2)
80	abs(hsa04962_1-hsa04962_2)
81	hsa00520_1+hsa00520_2
82	hsa05322_1+hsa05322_2
83	abs(hsa00770_1-hsa00770_2)

84	hsa00430_1+hsa00430_2
85	hsa05130_1+hsa05130_2
86	abs(hsa00730_1-hsa00730_2)
87	abs(hsa04140_1-hsa04140_2)
88	abs(hsa04710_1-hsa04710_2)
89	abs(hsa05412_1-hsa05412_2)
90	abs(hsa04630_1-hsa04630_2)
91	abs(hsa04740_1-hsa04740_2)
92	hsa04960_1+hsa04960_2
93	abs(hsa00524_1-hsa00524_2)
94	abs(hsa04120_1-hsa04120_2)
95	abs(hsa00603_1-hsa00603_2)
96	abs(hsa04720_1-hsa04720_2)
97	hsa05217_1+hsa05217_2
98	abs(hsa04512_1-hsa04512_2)
99	abs(hsa04966_1-hsa04966_2)
100	hsa00120_1+hsa00120_2
101	abs(hsa04141_1-hsa04141_2)
102	abs(hsa00563_1-hsa00563_2)
103	abs(hsa00790_1-hsa00790_2)
104	abs(hsa04640_1-hsa04640_2)
105	hsa04330_1+hsa04330_2
106	abs(hsa04974_1-hsa04974_2)
107	hsa02010_1+hsa02010_2
108	abs(hsa04650_1-hsa04650_2)
109	hsa00350_1+hsa00350_2
110	hsa00534_1+hsa00534_2
111	abs(hsa03022_1-hsa03022_2)
112	abs(hsa05310_1-hsa05310_2)
113	abs(hsa00130_1-hsa00130_2)
114	abs(hsa00290_1-hsa00290_2)
115	abs(hsa00780_1-hsa00780_2)
116	abs(hsa04970_1-hsa04970_2)
117	abs(hsa00920_1-hsa00920_2)
118	F5[e]
119	abs(hsa04530_1-hsa04530_2)
120	abs(hsa05220_1-hsa05220_2)
121	abs(hsa04142_1-hsa04142_2)
122	abs(hsa00650_1-hsa00650_2)
123	abs(hsa00480_1-hsa00480_2)
124	abs(hsa04742_1-hsa04742_2)
125	hsa00190_1+hsa00190_2
126	hsa05100_1+hsa05100_2

127	hsa00230_1+hsa00230_2
128	abs(hsa00052_1-hsa00052_2)
129	hsa04122_1+hsa04122_2
130	hsa05010_1+hsa05010_2
131	abs(hsa04621_1-hsa04621_2)
132	abs(hsa04114_1-hsa04114_2)
133	hsa04710_1+hsa04710_2
134	abs(hsa04130_1-hsa04130_2)
135	hsa00232_1+hsa00232_2
136	abs(hsa05131_1-hsa05131_2)
137	abs(hsa00531_1-hsa00531_2)
138	hsa00601_1+hsa00601_2
139	abs(hsa00450_1-hsa00450_2)
140	abs(hsa00532_1-hsa00532_2)
141	abs(hsa04145_1-hsa04145_2)
142	abs(hsa04360_1-hsa04360_2)
143	hsa00785_1+hsa00785_2
144	abs(hsa04144_1-hsa04144_2)
145	abs(hsa03050_1-hsa03050_2)
146	abs(hsa00982_1-hsa00982_2)
147	abs(hsa04914_1-hsa04914_2)
148	hsa04514_1+hsa04514_2
149	hsa04910_1+hsa04910_2
150	hsa00400_1+hsa00400_2
151	abs(hsa04610_1-hsa04610_2)
152	abs(hsa03410_1-hsa03410_2)
153	abs(hsa00190_1-hsa00190_2)
154	hsa00300_1+hsa00300_2
155	abs(hsa04623_1-hsa04623_2)
156	hsa00062_1+hsa00062_2
157	abs(hsa04020_1-hsa04020_2)
158	abs(hsa00601_1-hsa00601_2)
159	abs(hsa02010_1-hsa02010_2)
160	hsa00603_1+hsa00603_2
161	hsa04744_1+hsa04744_2
162	hsa00340_1+hsa00340_2
163	abs(hsa05222_1-hsa05222_2)
164	hsa03015_1+hsa03015_2
165	hsa00510_1+hsa00510_2
166	hsa00982_1+hsa00982_2
167	abs(hsa00280_1-hsa00280_2)
168	hsa00563_1+hsa00563_2
169	abs(hsa05414_1-hsa05414_2)

170	hsa04622_1+hsa04622_2
171	hsa04141_1+hsa04141_2
172	abs(hsa00534_1-hsa00534_2)
173	abs(hsa04666_1-hsa04666_2)
174	hsa04150_1+hsa04150_2
175	hsa00760_1+hsa00760_2
176	hsa04640_1+hsa04640_2
177	F4[d]
178	abs(hsa00051_1-hsa00051_2)
179	abs(hsa05323_1-hsa05323_2)
180	hsa04610_1+hsa04610_2
181	abs(hsa03060_1-hsa03060_2)
182	hsa05012_1+hsa05012_2
183	hsa04140_1+hsa04140_2
184	hsa00650_1+hsa00650_2
185	abs(hsa00514_1-hsa00514_2)
186	hsa00330_1+hsa00330_2
187	abs(hsa00604_1-hsa00604_2)
188	abs(hsa04122_1-hsa04122_2)
189	hsa04950_1+hsa04950_2
190	abs(hsa00471_1-hsa00471_2)
191	hsa04512_1+hsa04512_2
192	abs(hsa05143_1-hsa05143_2)
193	abs(hsa00240_1-hsa00240_2)
194	hsa00770_1+hsa00770_2
195	abs(hsa00460_1-hsa00460_2)
196	abs(hsa04916_1-hsa04916_2)
197	abs(hsa04810_1-hsa04810_2)
198	abs(hsa00100_1-hsa00100_2)
199	abs(hsa05332_1-hsa05332_2)
200	abs(hsa00640_1-hsa00640_2)
201	abs(hsa00230_1-hsa00230_2)
202	abs(hsa05410_1-hsa05410_2)
203	abs(hsa05340_1-hsa05340_2)
204	abs(hsa00591_1-hsa00591_2)
205	hsa00380_1+hsa00380_2
206	hsa03022_1+hsa03022_2
207	hsa04070_1+hsa04070_2
208	abs(hsa00120_1-hsa00120_2)
209	abs(hsa05214_1-hsa05214_2)
210	abs(hsa00533_1-hsa00533_2)
211	hsa00524_1+hsa00524_2
212	hsa00730_1+hsa00730_2

213	abs(hsa04612_1-hsa04612_2)
214	hsa04080_1+hsa04080_2
215	abs(hsa00750_1-hsa00750_2)
216	hsa04114_1+hsa04114_2
217	abs(hsa00500_1-hsa00500_2)
218	abs(hsa00511_1-hsa00511_2)
219	hsa03050_1+hsa03050_2
220	hsa04974_1+hsa04974_2
221	abs(hsa05416_1-hsa05416_2)
222	hsa05150_1+hsa05150_2
223	hsa00030_1+hsa00030_2
224	abs(hsa04730_1-hsa04730_2)
225	abs(hsa00350_1-hsa00350_2)
226	hsa04973_1+hsa04973_2
227	hsa04260_1+hsa04260_2
228	abs(hsa04062_1-hsa04062_2)
229	hsa01040_1+hsa01040_2
230	hsa03060_1+hsa03060_2
231	abs(hsa05014_1-hsa05014_2)
232	abs(hsa04930_1-hsa04930_2)
233	hsa00790_1+hsa00790_2
234	hsa03018_1+hsa03018_2
235	abs(hsa04672_1-hsa04672_2)
236	abs(hsa04510_1-hsa04510_2)
237	abs(hsa03030_1-hsa03030_2)
238	abs(hsa00330_1-hsa00330_2)
239	hsa00630_1+hsa00630_2
240	abs(hsa05218_1-hsa05218_2)
241	hsa00533_1+hsa00533_2
242	abs(hsa00600_1-hsa00600_2)
243	abs(hsa00062_1-hsa00062_2)
244	abs(hsa00512_1-hsa00512_2)
245	abs(hsa03015_1-hsa03015_2)
246	hsa05214_1+hsa05214_2
247	hsa00020_1+hsa00020_2
248	hsa00270_1+hsa00270_2
249	hsa05412_1+hsa05412_2
250	hsa00514_1+hsa00514_2
251	abs(hsa04350_1-hsa04350_2)
252	hsa00100_1+hsa00100_2
253	hsa03020_1+hsa03020_2
254	hsa00051_1+hsa00051_2
255	hsa00460_1+hsa00460_2

256	abs(hsa00340_1-hsa00340_2)
257	abs(hsa04971_1-hsa04971_2)
258	hsa00140_1+hsa00140_2
259	abs(hsa04664_1-hsa04664_2)
260	hsa00740_1+hsa00740_2
261	hsa00310_1+hsa00310_2
262	abs(hsa04260_1-hsa04260_2)
263	abs(hsa05320_1-hsa05320_2)
264	abs(hsa03440_1-hsa03440_2)
265	abs(hsa00030_1-hsa00030_2)
266	abs(hsa05144_1-hsa05144_2)
267	hsa00472_1+hsa00472_2
268	hsa04530_1+hsa04530_2
269	hsa00670_1+hsa00670_2
270	abs(hsa03020_1-hsa03020_2)
271	abs(hsa04920_1-hsa04920_2)
272	F7[g]
273	hsa00500_1+hsa00500_2
274	abs(hsa04010_1-hsa04010_2)
275	abs(hsa04972_1-hsa04972_2)
276	hsa00900_1+hsa00900_2
277	abs(hsa00010_1-hsa00010_2)
278	abs(hsa03013_1-hsa03013_2)
279	abs(hsa04060_1-hsa04060_2)
280	abs(hsa00360_1-hsa00360_2)
281	abs(hsa01040_1-hsa01040_2)
282	abs(hsa00670_1-hsa00670_2)
283	hsa04976_1+hsa04976_2
284	hsa05016_1+hsa05016_2
285	hsa04940_1+hsa04940_2
286	abs(hsa00562_1-hsa00562_2)
287	hsa05410_1+hsa05410_2
288	hsa04340_1+hsa04340_2
289	abs(hsa05100_1-hsa05100_2)
290	hsa03008_1+hsa03008_2
291	abs(hsa00040_1-hsa00040_2)
292	abs(hsa04146_1-hsa04146_2)
293	abs(hsa00020_1-hsa00020_2)
294	hsa05310_1+hsa05310_2
295	hsa00130_1+hsa00130_2
296	abs(hsa00590_1-hsa00590_2)
297	hsa00290_1+hsa00290_2
298	abs(hsa05330_1-hsa05330_2)

299	abs(hsa00250_1-hsa00250_2)
300	abs(hsa04520_1-hsa04520_2)
301	abs(hsa03008_1-hsa03008_2)
302	hsa00512_1+hsa00512_2
303	abs(hsa00380_1-hsa00380_2)
304	hsa03450_1+hsa03450_2
305	hsa00010_1+hsa00010_2
306	abs(hsa00900_1-hsa00900_2)
307	hsa00450_1+hsa00450_2
308	abs(hsa03320_1-hsa03320_2)
309	hsa00040_1+hsa00040_2
310	abs(hsa00740_1-hsa00740_2)
311	hsa04930_1+hsa04930_2
312	abs(hsa00564_1-hsa00564_2)
313	abs(hsa04110_1-hsa04110_2)
314	abs(hsa00970_1-hsa00970_2)
315	abs(hsa04622_1-hsa04622_2)
316	hsa00360_1+hsa00360_2
317	hsa04612_1+hsa04612_2
318	hsa00061_1+hsa00061_2
319	abs(hsa00620_1-hsa00620_2)
320	hsa04962_1+hsa04962_2
321	abs(hsa03040_1-hsa03040_2)
322	hsa00600_1+hsa00600_2
323	hsa00750_1+hsa00750_2
324	hsa04146_1+hsa04146_2
325	hsa05120_1+hsa05120_2
326	abs(hsa00053_1-hsa00053_2)
327	abs(hsa05219_1-hsa05219_2)
328	hsa04614_1+hsa04614_2
329	abs(hsa04912_1-hsa04912_2)
330	hsa05416_1+hsa05416_2
331	hsa00072_1+hsa00072_2
332	hsa05414_1+hsa05414_2
333	abs(hsa05145_1-hsa05145_2)
334	hsa04110_1+hsa04110_2
335	hsa00071_1+hsa00071_2
336	abs(hsa00980_1-hsa00980_2)
337	hsa04310_1+hsa04310_2
338	hsa00970_1+hsa00970_2
339	hsa00250_1+hsa00250_2
340	hsa03320_1+hsa03320_2
341	abs(hsa04012_1-hsa04012_2)

342	abs(hsa01100_1-hsa01100_2)
343	hsa00604_1+hsa00604_2
344	hsa03410_1+hsa03410_2
345	hsa05340_1+hsa05340_2
346	hsa04144_1+hsa04144_2
347	abs(hsa05216_1-hsa05216_2)
348	hsa00562_1+hsa00562_2
349	hsa00053_1+hsa00053_2
350	abs(hsa04270_1-hsa04270_2)
351	hsa03013_1+hsa03013_2
352	hsa00410_1+hsa00410_2
353	abs(hsa05211_1-hsa05211_2)
354	abs(hsa00071_1-hsa00071_2)
355	hsa04977_1+hsa04977_2
356	abs(hsa00565_1-hsa00565_2)
357	hsa00830_1+hsa00830_2
358	hsa00620_1+hsa00620_2
359	hsa03440_1+hsa03440_2
360	abs(hsa04660_1-hsa04660_2)
361	hsa05332_1+hsa05332_2
362	abs(hsa00260_1-hsa00260_2)
363	hsa04666_1+hsa04666_2
364	abs(hsa00860_1-hsa00860_2)
365	hsa00240_1+hsa00240_2
366	F6[f]
367	hsa03040_1+hsa03040_2
368	abs(hsa00072_1-hsa00072_2)
369	abs(hsa05142_1-hsa05142_2)
370	abs(hsa00410_1-hsa00410_2)
371	hsa05320_1+hsa05320_2
372	hsa04720_1+hsa04720_2
373	abs(hsa00561_1-hsa00561_2)
374	hsa04810_1+hsa04810_2
375	hsa04115_1+hsa04115_2
376	abs(hsa00592_1-hsa00592_2)
377	abs(hsa05223_1-hsa05223_2)
378	abs(hsa00983_1-hsa00983_2)
379	abs(hsa03420_1-hsa03420_2)
380	hsa05014_1+hsa05014_2
381	hsa04350_1+hsa04350_2
382	hsa00860_1+hsa00860_2
383	hsa00561_1+hsa00561_2
384	abs(hsa04722_1-hsa04722_2)

385	hsa00591_1+hsa00591_2
386	hsa04971_1+hsa04971_2
387	hsa04320_1+hsa04320_2
388	hsa03030_1+hsa03030_2
389	hsa04975_1+hsa04975_2
390	hsa05222_1+hsa05222_2
391	hsa00980_1+hsa00980_2
392	hsa04742_1+hsa04742_2
393	abs(hsa05200_1-hsa05200_2)
394	hsa00280_1+hsa00280_2
395	hsa05330_1+hsa05330_2
396	hsa05218_1+hsa05218_2
397	abs(hsa03430_1-hsa03430_2)
398	abs(hsa05140_1-hsa05140_2)
399	hsa01100_1+hsa01100_2
400	hsa04360_1+hsa04360_2
401	hsa00565_1+hsa00565_2
402	hsa04062_1+hsa04062_2
403	hsa00983_1+hsa00983_2
404	hsa00640_1+hsa00640_2
405	abs(hsa05160_1-hsa05160_2)
406	hsa04540_1+hsa04540_2
407	hsa00590_1+hsa00590_2
408	hsa00260_1+hsa00260_2
409	abs(hsa05213_1-hsa05213_2)
410	hsa04672_1+hsa04672_2
411	hsa04630_1+hsa04630_2
412	abs(hsa04662_1-hsa04662_2)
413	hsa03430_1+hsa03430_2
414	hsa04914_1+hsa04914_2
415	abs(hsa05210_1-hsa05210_2)
416	hsa04020_1+hsa04020_2
417	hsa04670_1+hsa04670_2
418	hsa00592_1+hsa00592_2
419	abs(hsa05212_1-hsa05212_2)
420	hsa03420_1+hsa03420_2
421	hsa04916_1+hsa04916_2
422	abs(hsa04620_1-hsa04620_2)
423	hsa00564_1+hsa00564_2
424	hsa04520_1+hsa04520_2
425	hsa05146_1+hsa05146_2
426	hsa04920_1+hsa04920_2
427	abs(hsa04210_1-hsa04210_2)

428	hsa04730_1+hsa04730_2
429	hsa04510_1+hsa04510_2
430	hsa04620_1+hsa04620_2
431	abs(hsa05221_1-hsa05221_2)
432	hsa04270_1+hsa04270_2
433	hsa05143_1+hsa05143_2
434	hsa05213_1+hsa05213_2
435	hsa05216_1+hsa05216_2
436	hsa04623_1+hsa04623_2
437	hsa05144_1+hsa05144_2
438	hsa05323_1+hsa05323_2
439	hsa04722_1+hsa04722_2
440	abs(hsa04380_1-hsa04380_2)
441	hsa04621_1+hsa04621_2
442	hsa04060_1+hsa04060_2
443	hsa05131_1+hsa05131_2
444	hsa04210_1+hsa04210_2
445	hsa05215_1+hsa05215_2
446	hsa04650_1+hsa04650_2
447	hsa05160_1+hsa05160_2
448	hsa05223_1+hsa05223_2
449	hsa04912_1+hsa04912_2
450	hsa05219_1+hsa05219_2
451	hsa04380_1+hsa04380_2
452	hsa04012_1+hsa04012_2
453	hsa05220_1+hsa05220_2
454	hsa04370_1+hsa04370_2
455	hsa05221_1+hsa05221_2
456	hsa05142_1+hsa05142_2
457	hsa04010_1+hsa04010_2
458	hsa05145_1+hsa05145_2
459	hsa04660_1+hsa04660_2
460	hsa05200_1+hsa05200_2
461	hsa05140_1+hsa05140_2
462	hsa04662_1+hsa04662_2
463	hsa05210_1+hsa05210_2
464	hsa04664_1+hsa04664_2
465	hsa05212_1+hsa05212_2

[a] The confidence score of two drugs in the combination, see “Chemical interaction” of Section “Features of drug combinations” for detail.

[b] The maximum confidence score between targets of drug d_1 and targets of d_2, see **Eq.** 1 and related text in Section “Features of drug combinations” for detail.

[c] The mean value of confidence scores between targets of drug d_1 and targets of d_2, see **Eq.** 1 and related text in Section "Features of drug combinations" for detail.

[d] The absolute value of difference of maximum confidence score between targets of drug d_1 and maximum confidence score between targets of drug d_2, see **Eq.** 2 and related text in Section "Features of drug combinations" for detail.

[e] The absolute value of difference of mean value of confidence scores between targets of drug d_1 and mean value of confidence score between targets of drug d_2, see **Eq.** 2 and related text in Section "Features of drug combinations" for detail.

[f] The sum of maximum confidence score between targets of drug d_1 and maximum confidence score between targets of drug d_2, see **Eq.** 2 and related text in Section "Features of drug combinations" for detail.

[g] The sum of mean value of confidence scores between targets of drug d_1 and mean value of confidence score between targets of drug d_2, see **Eq.** 2 and related text in Section "Features of drug combinations" for detail.

[h] The absolute value of the deference of enrichment score of drug d_1 and pathway hsa05215 ("Prostate cancer") and enrichment score of drug d_2 and pathway hsa05215 ("Prostate cancer"), see **Eq. 3** and related text in Section "Features of drug combinations" for detail.

[i] The sum of enrichment score of drug d_1 and pathway hsa04964 ("Proximal tubule bicarbonate reclamation") and enrichment score of drug d_2 and pathway hsa04964 ("Proximal tubule bicarbonate reclamation"), see **Eq. 3** and related text in Section "Features of drug combinations" for detail.

(III) Pathway ID *vs* Pathway name

Pathway ID	Pathway name
hsa00010	Glycolysis / Gluconeogenesis
hsa00020	Citrate cycle (TCA cycle)
hsa00030	Pentose phosphate pathway
hsa00040	Pentose and glucuronate interconversions
hsa00051	Fructose and mannose metabolism
hsa00052	Galactose metabolism
hsa00053	Ascorbate and aldarate metabolism
hsa00061	Fatty acid biosynthesis
hsa00062	Fatty acid elongation in mitochondria
hsa00071	Fatty acid metabolism
hsa00072	Synthesis and degradation of ketone bodies
hsa00100	Steroid biosynthesis
hsa00120	Primary bile acid biosynthesis
hsa00130	Ubiquinone and other terpenoid-quinone biosynthesis
hsa00140	Steroid hormone biosynthesis
hsa00190	Oxidative phosphorylation
hsa00230	Purine metabolism
hsa00232	Caffeine metabolism
hsa00240	Pyrimidine metabolism
hsa00250	Alanine, aspartate and glutamate metabolism
hsa00260	Glycine, serine and threonine metabolism
hsa00270	Cysteine and methionine metabolism
hsa00280	Valine, leucine and isoleucine degradation
hsa00290	Valine, leucine and isoleucine biosynthesis
hsa00300	Lysine biosynthesis
hsa00310	Lysine degradation
hsa00330	Arginine and proline metabolism
hsa00340	Histidine metabolism
hsa00350	Tyrosine metabolism
hsa00360	Phenylalanine metabolism
hsa00380	Tryptophan metabolism
hsa00400	Phenylalanine, tyrosine and tryptophan biosynthesis
hsa00410	beta-Alanine metabolism
hsa00430	Taurine and hypotaurine metabolism
hsa00450	Selenocompound metabolism
hsa00460	Cyanoamino acid metabolism
hsa00471	D-Glutamine and D-glutamate metabolism
hsa00472	D-Arginine and D-ornithine metabolism
hsa00480	Glutathione metabolism
hsa00500	Starch and sucrose metabolism

hsa00510	N-Glycan biosynthesis
hsa00511	Other glycan degradation
hsa00512	Mucin type O-Glycan biosynthesis
hsa00514	Other types of O-glycan biosynthesis
hsa00520	Amino sugar and nucleotide sugar metabolism
hsa00524	Butirosin and neomycin biosynthesis
hsa00531	Glycosaminoglycan degradation
hsa00532	Glycosaminoglycan biosynthesis - chondroitin sulfate
hsa00533	Glycosaminoglycan biosynthesis - keratan sulfate
hsa00534	Glycosaminoglycan biosynthesis - heparan sulfate
hsa00561	Glycerolipid metabolism
hsa00562	Inositol phosphate metabolism
hsa00563	Glycosylphosphatidylinositol(GPI)-anchor biosynthesis
hsa00564	Glycerophospholipid metabolism
hsa00565	Ether lipid metabolism
hsa00590	Arachidonic acid metabolism
hsa00591	Linoleic acid metabolism
hsa00592	alpha-Linolenic acid metabolism
hsa00600	Sphingolipid metabolism
hsa00601	Glycosphingolipid biosynthesis - lacto and neolacto series
hsa00603	Glycosphingolipid biosynthesis - globo series
hsa00604	Glycosphingolipid biosynthesis - ganglio series
hsa00620	Pyruvate metabolism
hsa00630	Glyoxylate and dicarboxylate metabolism
hsa00640	Propanoate metabolism
hsa00650	Butanoate metabolism
hsa00670	One carbon pool by folate
hsa00730	Thiamine metabolism
hsa00740	Riboflavin metabolism
hsa00750	Vitamin B6 metabolism
hsa00760	Nicotinate and nicotinamide metabolism
hsa00770	Pantothenate and CoA biosynthesis
hsa00780	Biotin metabolism
hsa00785	Lipoic acid metabolism
hsa00790	Folate biosynthesis
hsa00830	Retinol metabolism
hsa00860	Porphyrin and chlorophyll metabolism
hsa00900	Terpenoid backbone biosynthesis
hsa00910	Nitrogen metabolism
hsa00920	Sulfur metabolism
hsa00970	Aminoacyl-tRNA biosynthesis
hsa00980	Metabolism of xenobiotics by cytochrome P450
hsa00982	Drug metabolism - cytochrome P450

hsa00983	Drug metabolism - other enzymes
hsa01040	Biosynthesis of unsaturated fatty acids
hsa01100	Metabolic pathways
hsa02010	ABC transporters
hsa03008	Ribosome biogenesis in eukaryotes
hsa03010	Ribosome
hsa03013	RNA transport
hsa03015	mRNA surveillance pathway
hsa03018	RNA degradation
hsa03020	RNA polymerase
hsa03022	Basal transcription factors
hsa03030	DNA replication
hsa03040	Spliceosome
hsa03050	Proteasome
hsa03060	Protein export
hsa03320	PPAR signaling pathway
hsa03410	Base excision repair
hsa03420	Nucleotide excision repair
hsa03430	Mismatch repair
hsa03440	Homologous recombination
hsa03450	Non-homologous end-joining
hsa04010	MAPK signaling pathway
hsa04012	ErbB signaling pathway
hsa04020	Calcium signaling pathway
hsa04060	Cytokine-cytokine receptor interaction
hsa04062	Chemokine signaling pathway
hsa04070	Phosphatidylinositol signaling system
hsa04080	Neuroactive ligand-receptor interaction
hsa04110	Cell cycle
hsa04114	Oocyte meiosis
hsa04115	p53 signaling pathway
hsa04120	Ubiquitin mediated proteolysis
hsa04122	Sulfur relay system
hsa04130	SNARE interactions in vesicular transport
hsa04140	Regulation of autophagy
hsa04141	Protein processing in endoplasmic reticulum
hsa04142	Lysosome
hsa04144	Endocytosis
hsa04145	Phagosome
hsa04146	Peroxisome
hsa04150	mTOR signaling pathway
hsa04210	Apoptosis
hsa04260	Cardiac muscle contraction

hsa04270	Vascular smooth muscle contraction
hsa04310	Wnt signaling pathway
hsa04320	Dorso-ventral axis formation
hsa04330	Notch signaling pathway
hsa04340	Hedgehog signaling pathway
hsa04350	TGF-beta signaling pathway
hsa04360	Axon guidance
hsa04370	VEGF signaling pathway
hsa04380	Osteoclast differentiation
hsa04510	Focal adhesion
hsa04512	ECM-receptor interaction
hsa04514	Cell adhesion molecules (CAMs)
hsa04520	Adherens junction
hsa04530	Tight junction
hsa04540	Gap junction
hsa04610	Complement and coagulation cascades
hsa04612	Antigen processing and presentation
hsa04614	Renin-angiotensin system
hsa04620	Toll-like receptor signaling pathway
hsa04621	NOD-like receptor signaling pathway
hsa04622	RIG-I-like receptor signaling pathway
hsa04623	Cytosolic DNA-sensing pathway
hsa04630	Jak-STAT signaling pathway
hsa04640	Hematopoietic cell lineage
hsa04650	Natural killer cell mediated cytotoxicity
hsa04660	T cell receptor signaling pathway
hsa04662	B cell receptor signaling pathway
hsa04664	Fc epsilon RI signaling pathway
hsa04666	Fc gamma R-mediated phagocytosis
hsa04670	Leukocyte transendothelial migration
hsa04672	Intestinal immune network for IgA production
hsa04710	Circadian rhythm - mammal
hsa04720	Long-term potentiation
hsa04722	Neurotrophin signaling pathway
hsa04730	Long-term depression
hsa04740	Olfactory transduction
hsa04742	Taste transduction
hsa04744	Phototransduction
hsa04810	Regulation of actin cytoskeleton
hsa04910	Insulin signaling pathway
hsa04912	GnRH signaling pathway
hsa04914	Progesterone-mediated oocyte maturation
hsa04916	Melanogenesis

hsa04920	Adipocytokine signaling pathway
hsa04930	Type II diabetes mellitus
hsa04940	Type I diabetes mellitus
hsa04950	Maturity onset diabetes of the young
hsa04960	Aldosterone-regulated sodium reabsorption
hsa04962	Vasopressin-regulated water reabsorption
hsa04964	Proximal tubule bicarbonate reclamation
hsa04966	Collecting duct acid secretion
hsa04970	Salivary secretion
hsa04971	Gastric acid secretion
hsa04972	Pancreatic secretion
hsa04973	Carbohydrate digestion and absorption
hsa04974	Protein digestion and absorption
hsa04975	Fat digestion and absorption
hsa04976	Bile secretion
hsa04977	Vitamin digestion and absorption
hsa05010	Alzheimer's disease
hsa05012	Parkinson's disease
hsa05014	Amyotrophic lateral sclerosis (ALS)
hsa05016	Huntington's disease
hsa05020	Prion diseases
hsa05100	Bacterial invasion of epithelial cells
hsa05110	Vibrio cholerae infection
hsa05120	Epithelial cell signaling in Helicobacter pylori infection
hsa05130	Pathogenic Escherichia coli infection
hsa05131	Shigellosis
hsa05140	Leishmaniasis
hsa05142	Chagas disease (American trypanosomiasis)
hsa05143	African trypanosomiasis
hsa05144	Malaria
hsa05145	Toxoplasmosis
hsa05146	Amoebiasis
hsa05150	Staphylococcus aureus infection
hsa05160	Hepatitis C
hsa05200	Pathways in cancer
hsa05210	Colorectal cancer
hsa05211	Renal cell carcinoma
hsa05212	Pancreatic cancer
hsa05213	Endometrial cancer
hsa05214	Glioma
hsa05215	Prostate cancer
hsa05216	Thyroid cancer
hsa05217	Basal cell carcinoma

hsa05218	Melanoma
hsa05219	Bladder cancer
hsa05220	Chronic myeloid leukemia
hsa05221	Acute myeloid leukemia
hsa05222	Small cell lung cancer
hsa05223	Non-small cell lung cancer
hsa05310	Asthma
hsa05320	Autoimmune thyroid disease
hsa05322	Systemic lupus erythematosus
hsa05323	Rheumatoid arthritis
hsa05330	Allograft rejection
hsa05332	Graft-versus-host disease
hsa05340	Primary immunodeficiency
hsa05410	Hypertrophic cardiomyopathy (HCM)
hsa05412	Arrhythmogenic right ventricular cardiomyopathy (ARVC)
hsa05414	Dilated cardiomyopathy
hsa05416	Viral myocarditis
