# Supplementary material for: Prediction of Effective Drug Combinations by Chemical Interaction, Protein Interaction and Target Enrichment of KEGG Pathways
Source: Biomed Res Int. 2013 Sep 5;2013:723780. doi: 10.1155/2013/723780 (PMC3780555; doi:10.1155/2013/723780)
Supplement: Supplementary file 4 [file 723780.f4.pdf]

Supplementary Material IV: Detailed data in IFS curve and AUC scores

Number of features	Sensitivity (SN)	Specificity (SP)	Prediction accuracy (ACC)	Matthews's correlation coefficient (MCC)	AUC score
4	0.6694	0.9372	0.8926	0.6107	0.8116
5	0.6777	0.9488	0.9036	0.644	0.8156
6	0.6281	0.9455	0.8926	0.5984	0.8286
7	0.686	0.9455	0.9022	0.6422	0.8622
8	0.6942	0.9455	0.9036	0.6484	0.8509
9	0.686	0.9455	0.9022	0.6422	0.8602
10	0.6612	0.9521	0.9036	0.6398	0.8539
11	0.6364	0.9421	0.8912	0.5969	0.8523
12	0.6364	0.9537	0.9008	0.6252	0.8802
13	0.6446	0.957	0.905	0.64	0.8753
14	0.6446	0.9455	0.8953	0.6111	0.8684
15	0.6777	0.9421	0.8981	0.6283	0.8616
16	0.6446	0.9471	0.8967	0.6151	0.8754
17	0.6694	0.9471	0.9008	0.6338	0.8861
18	0.6529	0.9521	0.9022	0.6336	0.8684
19	0.6198	0.9521	0.8967	0.6084	0.8847
20	0.6281	0.9521	0.8981	0.6147	0.8659
21	0.6364	0.9504	0.8981	0.6169	0.8583
22	0.6364	0.9488	0.8967	0.6128	0.8562
23	0.6281	0.9471	0.8939	0.6024	0.8781
24	0.6116	0.9521	0.8953	0.602	0.8573
25	0.595	0.9554	0.8953	0.5977	0.8809
26	0.6033	0.957	0.8981	0.6085	0.8606
27	0.595	0.9554	0.8953	0.5977	0.8639
28	0.6116	0.9488	0.8926	0.5937	0.8672
29	0.6033	0.9488	0.8912	0.5873	0.8501
30	0.6446	0.9554	0.9036	0.6357	0.8509
31	0.6694	0.957	0.9091	0.6586	0.8860
32	0.6364	0.962	0.9077	0.647	0.8773
33	0.6198	0.9537	0.8981	0.6126	0.8455
34	0.5702	0.957	0.8926	0.5827	0.8784
35	0.5785	0.9554	0.8926	0.5848	0.8614
36	0.5785	0.9587	0.8953	0.5936	0.8497
37	0.5537	0.962	0.8939	0.5832	0.8716
38	0.6033	0.962	0.9022	0.6219	0.8507
39	0.6364	0.9471	0.8953	0.6087	0.9005

40	0.6364	0.9488	0.8967	0.6128	0.8704
41	0.6198	0.9636	0.9063	0.6391	0.8864
42	0.6116	0.9521	0.8953	0.602	0.8815
43	0.6116	0.9587	0.9008	0.6193	0.8355
44	0.6281	0.9554	0.9008	0.6232	0.8403
45	0.6198	0.9554	0.8994	0.6169	0.8492
46	0.5702	0.9587	0.8939	0.5871	0.8476
47	0.5785	0.9686	0.9036	0.6217	0.8690
48	0.5868	0.9603	0.8981	0.6046	0.8588
49	0.5537	0.9554	0.8884	0.5651	0.8341
50	0.5785	0.9636	0.8994	0.6074	0.8716
51	0.562	0.9719	0.9036	0.6188	0.8617
52	0.5868	0.9471	0.8871	0.5702	0.8713
53	0.5455	0.9521	0.8843	0.5498	0.8639
54	0.595	0.9521	0.8926	0.5892	0.8228
55	0.6529	0.9669	0.9146	0.6731	0.8803
56	0.5537	0.962	0.8939	0.5832	0.8546
57	0.6033	0.957	0.8981	0.6085	0.8602
58	0.6033	0.9603	0.9008	0.6174	0.8586
59	0.595	0.9669	0.905	0.6296	0.8412
60	0.5124	0.962	0.8871	0.5499	0.8314
61	0.5537	0.962	0.8939	0.5832	0.8391
62	0.5455	0.9537	0.8857	0.5541	0.8607
63	0.5455	0.9669	0.8967	0.5909	0.8765
64	0.6116	0.957	0.8994	0.6149	0.8772
65	0.595	0.9719	0.9091	0.6442	0.8646
66	0.5455	0.9669	0.8967	0.5909	0.8692
67	0.5868	0.9554	0.8939	0.5913	0.8499
68	0.5702	0.9521	0.8884	0.5697	0.8496
69	0.5041	0.9669	0.8898	0.5576	0.8648
70	0.5289	0.9521	0.8815	0.5364	0.8547
71	0.5702	0.9537	0.8898	0.5739	0.8511
72	0.5785	0.9537	0.8912	0.5805	0.8631
73	0.6281	0.9554	0.9008	0.6232	0.8687
74	0.5455	0.9636	0.8939	0.5813	0.8732
75	0.5868	0.9636	0.9008	0.6138	0.8645
76	0.5702	0.9603	0.8953	0.5917	0.8566
77	0.5455	0.9636	0.8939	0.5813	0.8209
78	0.5207	0.9603	0.8871	0.5519	0.8562

79	0.5537	0.9653	0.8967	0.5926	0.8457
80	0.4793	0.9636	0.8829	0.5272	0.8379
81	0.5041	0.9603	0.8843	0.5384	0.8584
82	0.5289	0.9636	0.8912	0.5681	0.8441
83	0.5207	0.9603	0.8871	0.5519	0.8504
84	0.5372	0.9587	0.8884	0.5608	0.8364
85	0.5785	0.9686	0.9036	0.6217	0.8562
86	0.4876	0.9421	0.8664	0.4771	0.8480
87	0.5702	0.9587	0.8939	0.5871	0.8689
88	0.4793	0.9587	0.8788	0.513	0.8313
89	0.4876	0.9587	0.8802	0.52	0.8368
90	0.5041	0.962	0.8857	0.5431	0.8474
91	0.5289	0.9603	0.8884	0.5587	0.8601
92	0.4959	0.962	0.8843	0.5362	0.8468
93	0.5537	0.962	0.8939	0.5832	0.8484
94	0.5207	0.9554	0.8829	0.5383	0.8225
95	0.4711	0.9587	0.8774	0.506	0.8309
96	0.5537	0.9702	0.9008	0.6073	0.8377
97	0.5455	0.9636	0.8939	0.5813	0.8588
98	0.5207	0.9521	0.8802	0.5296	0.8178
99	0.5124	0.9636	0.8884	0.5546	0.8377
100	0.5455	0.9603	0.8912	0.572	0.8422
101	0.5455	0.9521	0.8843	0.5498	0.8539
102	0.5289	0.9521	0.8815	0.5364	0.8281
103	0.562	0.9587	0.8926	0.5806	0.8369
104	0.5041	0.9636	0.8871	0.5478	0.8542
105	0.5207	0.957	0.8843	0.5428	0.8148
106	0.5785	0.962	0.8981	0.6027	0.8311
107	0.5124	0.9587	0.8843	0.5406	0.8400
108	0.5289	0.957	0.8857	0.5496	0.8065
109	0.5207	0.962	0.8884	0.5566	0.8528
110	0.5124	0.9636	0.8884	0.5546	0.8433
111	0.4793	0.9686	0.8871	0.5421	0.8204
112	0.5124	0.9603	0.8857	0.5452	0.8064
113	0.4959	0.9603	0.8829	0.5315	0.8287
114	0.5041	0.9587	0.8829	0.5337	0.8032
115	0.5041	0.9587	0.8829	0.5337	0.8223
116	0.5207	0.9504	0.8788	0.5253	0.8192
117	0.4876	0.9603	0.8815	0.5246	0.8398

118	0.5289	0.9537	0.8829	0.5407	0.8096
119	0.4711	0.962	0.8802	0.5154	0.7855
120	0.5124	0.9669	0.8912	0.5644	0.8237
121	0.4793	0.9603	0.8802	0.5177	0.8023
122	0.5041	0.9521	0.8774	0.5159	0.8425
123	0.4959	0.962	0.8843	0.5362	0.8428
124	0.5372	0.9587	0.8884	0.5608	0.8397
125	0.4876	0.9603	0.8815	0.5246	0.7988
126	0.4628	0.957	0.8747	0.4943	0.8114
127	0.5289	0.9669	0.8939	0.5777	0.8470
128	0.4876	0.9537	0.876	0.5064	0.7780
129	0.4711	0.9736	0.8898	0.551	0.8042
130	0.4876	0.9603	0.8815	0.5246	0.8348
131	0.4793	0.9603	0.8802	0.5177	0.8396
132	0.5455	0.9636	0.8939	0.5813	0.8493
133	0.5289	0.9653	0.8926	0.5729	0.8288
134	0.5124	0.962	0.8871	0.5499	0.8198
135	0.5124	0.9521	0.8788	0.5228	0.8474
136	0.4793	0.9702	0.8884	0.5473	0.7918
137	0.4959	0.9537	0.8774	0.5134	0.8227
138	0.4545	0.9636	0.8788	0.5061	0.8170
139	0.5537	0.9554	0.8884	0.5651	0.8160
140	0.5455	0.9636	0.8939	0.5813	0.8258
141	0.4959	0.9603	0.8829	0.5315	0.8083
142	0.5041	0.9686	0.8912	0.5626	0.8161
143	0.5537	0.9686	0.8994	0.6024	0.8238
144	0.5124	0.9471	0.8747	0.5101	0.8061
145	0.5537	0.9702	0.9008	0.6073	0.8381
146	0.5041	0.962	0.8857	0.5431	0.8176
147	0.5289	0.9603	0.8884	0.5587	0.8025
148	0.4711	0.9603	0.8788	0.5107	0.8101
149	0.5041	0.957	0.8815	0.5292	0.8335
150	0.4959	0.9636	0.8857	0.541	0.8471
151	0.4876	0.9603	0.8815	0.5246	0.8012
152	0.5207	0.9554	0.8829	0.5383	0.8226
153	0.5207	0.9603	0.8871	0.5519	0.8038
154	0.4876	0.9653	0.8857	0.539	0.8013
155	0.5124	0.9686	0.8926	0.5694	0.8240
156	0.5041	0.9537	0.8788	0.5203	0.8017

157	0.5289	0.9669	0.8939	0.5777	0.8321
158	0.4876	0.957	0.8788	0.5154	0.8207
159	0.5041	0.9636	0.8871	0.5478	0.8284
160	0.5041	0.9669	0.8898	0.5576	0.8409
161	0.4545	0.9702	0.8843	0.5264	0.7927
162	0.5124	0.957	0.8829	0.536	0.8075
163	0.4711	0.9554	0.8747	0.4969	0.8056
164	0.4876	0.9587	0.8802	0.52	0.8337
165	0.4876	0.962	0.8829	0.5293	0.7906
166	0.5041	0.9587	0.8829	0.5337	0.8193
167	0.5207	0.962	0.8884	0.5566	0.8127
168	0.4628	0.9653	0.8815	0.5181	0.8154
169	0.4959	0.962	0.8843	0.5362	0.8303
170	0.5289	0.957	0.8857	0.5496	0.8255
171	0.4711	0.9521	0.8719	0.488	0.7476
172	0.5207	0.9537	0.8815	0.5339	0.8393
173	0.4959	0.9669	0.8884	0.5508	0.8112
174	0.4711	0.9603	0.8788	0.5107	0.8173
175	0.5124	0.962	0.8871	0.5499	0.8259
176	0.4876	0.9537	0.876	0.5064	0.7990
177	0.4545	0.957	0.8733	0.4872	0.8207
178	0.4628	0.9587	0.876	0.4989	0.7648
179	0.4959	0.9752	0.8953	0.5768	0.8184
180	0.4959	0.957	0.8802	0.5223	0.8238
181	0.5041	0.9471	0.8733	0.5032	0.8161
182	0.4628	0.9653	0.8815	0.5181	0.8101
183	0.4876	0.962	0.8829	0.5293	0.8126
184	0.4711	0.9603	0.8788	0.5107	0.7751
185	0.4628	0.9636	0.8802	0.5132	0.8014
186	0.4711	0.9587	0.8774	0.506	0.8051
187	0.5124	0.9554	0.8815	0.5315	0.7956
188	0.4793	0.9603	0.8802	0.5177	0.8229
189	0.4628	0.9669	0.8829	0.5231	0.8023
190	0.4711	0.962	0.8802	0.5154	0.7906
191	0.4545	0.9636	0.8788	0.5061	0.7898
192	0.4876	0.9702	0.8898	0.5542	0.8167
193	0.4545	0.9653	0.8802	0.5111	0.8167
194	0.4793	0.962	0.8815	0.5224	0.8277
195	0.5124	0.9636	0.8884	0.5546	0.7960

196	0.5289	0.9702	0.8967	0.5877	0.8107
197	0.4711	0.9736	0.8898	0.551	0.7959
198	0.4793	0.962	0.8815	0.5224	0.8163
199	0.4793	0.9702	0.8884	0.5473	0.8011
200	0.4628	0.9603	0.8774	0.5036	0.7660
201	0.4463	0.9653	0.8788	0.504	0.8004
202	0.4876	0.9653	0.8857	0.539	0.8303
203	0.4793	0.9719	0.8898	0.5525	0.7943
204	0.5041	0.9702	0.8926	0.5677	0.8475
205	0.4545	0.9603	0.876	0.4965	0.7930
206	0.5207	0.9603	0.8871	0.5519	0.7997
207	0.4545	0.9636	0.8788	0.5061	0.7529
208	0.4876	0.9653	0.8857	0.539	0.8011
209	0.4628	0.9769	0.8912	0.5551	0.8111
210	0.4628	0.9636	0.8802	0.5132	0.8052
211	0.5207	0.9636	0.8898	0.5614	0.8039
212	0.4463	0.9587	0.8733	0.4846	0.7856
213	0.5124	0.9669	0.8912	0.5644	0.8187
214	0.4793	0.9603	0.8802	0.5177	0.7984
215	0.4463	0.9686	0.8815	0.5141	0.7775
216	0.4215	0.9669	0.876	0.4873	0.7647
217	0.4793	0.9702	0.8884	0.5473	0.7843
218	0.4711	0.9653	0.8829	0.5252	0.7918
219	0.4959	0.962	0.8843	0.5362	0.8346
220	0.4793	0.9702	0.8884	0.5473	0.8136
221	0.4711	0.9603	0.8788	0.5107	0.7605
222	0.4793	0.9636	0.8829	0.5272	0.8142
223	0.4463	0.9636	0.8774	0.499	0.7954
224	0.4628	0.957	0.8747	0.4943	0.7646
225	0.4711	0.9669	0.8843	0.5301	0.7826
226	0.438	0.9669	0.8788	0.5018	0.7882
227	0.4876	0.9636	0.8843	0.5341	0.8075
228	0.4298	0.962	0.8733	0.4797	0.8106
229	0.5041	0.9669	0.8898	0.5576	0.7973
230	0.5041	0.9603	0.8843	0.5384	0.7578
231	0.4711	0.9587	0.8774	0.506	0.7987
232	0.4959	0.9686	0.8898	0.5558	0.8223
233	0.4545	0.9587	0.8747	0.4918	0.7736
234	0.4793	0.9669	0.8857	0.5371	0.7831

235	0.5124	0.9587	0.8843	0.5406	0.8214
236	0.4711	0.9653	0.8829	0.5252	0.7971
237	0.4545	0.9653	0.8802	0.5111	0.7968
238	0.4628	0.9636	0.8802	0.5132	0.7775
239	0.5207	0.9636	0.8898	0.5614	0.8046
240	0.4793	0.9603	0.8802	0.5177	0.8038
241	0.5207	0.957	0.8843	0.5428	0.8232
242	0.4711	0.957	0.876	0.5014	0.7842
243	0.4628	0.9636	0.8802	0.5132	0.7995
244	0.4793	0.9702	0.8884	0.5473	0.7812
245	0.4298	0.9752	0.8843	0.5214	0.7626
246	0.5207	0.9587	0.8857	0.5473	0.7854
247	0.4711	0.9587	0.8774	0.506	0.8028
248	0.4711	0.9603	0.8788	0.5107	0.7905
249	0.4793	0.9636	0.8829	0.5272	0.7709
250	0.4132	0.9636	0.8719	0.4699	0.7835
251	0.4876	0.9521	0.8747	0.502	0.7858
252	0.4628	0.9653	0.8815	0.5181	0.7598
253	0.4876	0.9702	0.8898	0.5542	0.7971
254	0.4793	0.9554	0.876	0.5039	0.7558
255	0.4628	0.9554	0.8733	0.4898	0.7963
256	0.438	0.962	0.8747	0.4869	0.7486
257	0.4793	0.9587	0.8788	0.513	0.7614
258	0.4959	0.962	0.8843	0.5362	0.7961
259	0.4793	0.962	0.8815	0.5224	0.7979
260	0.4298	0.9603	0.8719	0.4749	0.8050
261	0.4463	0.9587	0.8733	0.4846	0.7924
262	0.4876	0.962	0.8829	0.5293	0.8037
263	0.438	0.9587	0.8719	0.4774	0.7808
264	0.4628	0.9603	0.8774	0.5036	0.8141
265	0.4793	0.9669	0.8857	0.5371	0.7806
266	0.4959	0.9669	0.8884	0.5508	0.8166
267	0.4711	0.962	0.8802	0.5154	0.7557
268	0.4628	0.9636	0.8802	0.5132	0.8213
269	0.4793	0.957	0.8774	0.5084	0.7710
270	0.4298	0.9554	0.8678	0.4609	0.7722
271	0.4876	0.9636	0.8843	0.5341	0.7858
272	0.4628	0.9719	0.8871	0.5387	0.7733
273	0.4628	0.957	0.8747	0.4943	0.8007

274	0.4959	0.9554	0.8788	0.5178	0.8056
275	0.4545	0.9702	0.8843	0.5264	0.7950
276	0.4628	0.962	0.8788	0.5084	0.7695
277	0.4711	0.962	0.8802	0.5154	0.7734
278	0.4793	0.9669	0.8857	0.5371	0.7830
279	0.4628	0.9653	0.8815	0.5181	0.7607
280	0.4628	0.957	0.8747	0.4943	0.7637
281	0.4959	0.9587	0.8815	0.5269	0.8150
282	0.4793	0.9554	0.876	0.5039	0.7804
283	0.4793	0.9686	0.8871	0.5421	0.8114
284	0.4793	0.962	0.8815	0.5224	0.7609
285	0.4545	0.9719	0.8857	0.5317	0.8017
286	0.4298	0.9669	0.8774	0.4946	0.7363
287	0.4628	0.9686	0.8843	0.5282	0.8053
288	0.4545	0.9653	0.8802	0.5111	0.7932
289	0.4876	0.962	0.8829	0.5293	0.8092
290	0.4545	0.9587	0.8747	0.4918	0.7489
291	0.4628	0.962	0.8788	0.5084	0.7823
292	0.4628	0.9603	0.8774	0.5036	0.7785
293	0.4793	0.957	0.8774	0.5084	0.8193
294	0.4793	0.9603	0.8802	0.5177	0.7829
295	0.4298	0.9669	0.8774	0.4946	0.7858
296	0.4793	0.9587	0.8788	0.513	0.8118
297	0.4628	0.9719	0.8871	0.5387	0.7719
298	0.4793	0.9537	0.8747	0.4994	0.7882
299	0.4463	0.9686	0.8815	0.5141	0.7550
300	0.4545	0.9603	0.876	0.4965	0.7774
301	0.4711	0.9587	0.8774	0.506	0.7702
302	0.5041	0.9669	0.8898	0.5576	0.8213
303	0.4545	0.9554	0.8719	0.4826	0.7528
304	0.4793	0.9636	0.8829	0.5272	0.7757
305	0.4628	0.9653	0.8815	0.5181	0.7990
306	0.4959	0.9686	0.8898	0.5558	0.7891
307	0.4876	0.9636	0.8843	0.5341	0.7869
308	0.4711	0.9603	0.8788	0.5107	0.7731
309	0.4463	0.962	0.876	0.4941	0.7517
310	0.5124	0.9603	0.8857	0.5452	0.7789
311	0.4959	0.9669	0.8884	0.5508	0.8267
312	0.5041	0.9653	0.8884	0.5527	0.8126

313	0.4711	0.9686	0.8857	0.5352	0.8040
314	0.4463	0.9653	0.8788	0.504	0.7812
315	0.438	0.957	0.8705	0.4728	0.7734
316	0.4793	0.962	0.8815	0.5224	0.7648
317	0.4628	0.9636	0.8802	0.5132	0.7819
318	0.4545	0.9636	0.8788	0.5061	0.7644
319	0.4959	0.962	0.8843	0.5362	0.7903
320	0.4876	0.9702	0.8898	0.5542	0.7973
321	0.4793	0.9587	0.8788	0.513	0.7910
322	0.4545	0.9669	0.8815	0.5161	0.8138
323	0.4298	0.9653	0.876	0.4895	0.7746
324	0.4793	0.9554	0.876	0.5039	0.7657
325	0.4876	0.9653	0.8857	0.539	0.7863
326	0.4463	0.9636	0.8774	0.499	0.7612
327	0.4793	0.9554	0.876	0.5039	0.7842
328	0.4628	0.9686	0.8843	0.5282	0.7990
329	0.5207	0.9653	0.8912	0.5662	0.7744
330	0.4711	0.9636	0.8815	0.5202	0.7933
331	0.4711	0.9686	0.8857	0.5352	0.7656
332	0.4298	0.9636	0.8747	0.4846	0.7686
333	0.5041	0.9554	0.8802	0.5247	0.7835
334	0.4959	0.9587	0.8815	0.5269	0.8154
335	0.4545	0.9686	0.8829	0.5212	0.7910
336	0.4959	0.962	0.8843	0.5362	0.8126
337	0.4463	0.9636	0.8774	0.499	0.7859
338	0.4545	0.962	0.8774	0.5013	0.7658
339	0.4545	0.957	0.8733	0.4872	0.7967
340	0.4876	0.9636	0.8843	0.5341	0.7988
341	0.4628	0.957	0.8747	0.4943	0.7722
342	0.4628	0.9603	0.8774	0.5036	0.7956
343	0.4545	0.9669	0.8815	0.5161	0.7395
344	0.405	0.962	0.8691	0.4575	0.8103
345	0.4959	0.9636	0.8857	0.541	0.7812
346	0.4463	0.9653	0.8788	0.504	0.7504
347	0.4711	0.9636	0.8815	0.5202	0.7312
348	0.4711	0.9603	0.8788	0.5107	0.7591
349	0.4711	0.9554	0.8747	0.4969	0.7534
350	0.4545	0.9653	0.8802	0.5111	0.7291
351	0.4793	0.962	0.8815	0.5224	0.7377

352	0.4463	0.9653	0.8788	0.504	0.7963
353	0.4876	0.9603	0.8815	0.5246	0.7845
354	0.4959	0.9719	0.8926	0.5662	0.7629
355	0.5124	0.9504	0.8774	0.5185	0.8158
356	0.4463	0.9603	0.8747	0.4893	0.7576
357	0.4711	0.957	0.876	0.5014	0.7338
358	0.4793	0.9636	0.8829	0.5272	0.8033
359	0.4298	0.962	0.8733	0.4797	0.7262
360	0.4711	0.9587	0.8774	0.506	0.7942
361	0.4628	0.9653	0.8815	0.5181	0.7811
362	0.4959	0.9702	0.8912	0.561	0.8182
363	0.4793	0.9537	0.8747	0.4994	0.7600
364	0.4545	0.9686	0.8829	0.5212	0.7747
365	0.4876	0.9537	0.876	0.5064	0.7779
366	0.4711	0.9653	0.8829	0.5252	0.7423
367	0.4876	0.962	0.8829	0.5293	0.7797
368	0.4711	0.9554	0.8747	0.4969	0.8130
369	0.4628	0.9603	0.8774	0.5036	0.7625
370	0.4876	0.9504	0.8733	0.4977	0.7396
371	0.4876	0.9653	0.8857	0.539	0.8166
372	0.4876	0.9653	0.8857	0.539	0.7585
373	0.4711	0.9587	0.8774	0.506	0.7543
374	0.4215	0.9669	0.876	0.4873	0.7746
375	0.3967	0.9554	0.8623	0.4311	0.7201
376	0.4876	0.9636	0.8843	0.5341	0.7638
377	0.438	0.9636	0.876	0.4918	0.7837
378	0.4545	0.9554	0.8719	0.4826	0.7545
379	0.4876	0.9669	0.8871	0.544	0.8007
380	0.4793	0.962	0.8815	0.5224	0.7991
381	0.4545	0.9636	0.8788	0.5061	0.7394
382	0.4628	0.962	0.8788	0.5084	0.7741
383	0.4959	0.9669	0.8884	0.5508	0.8143
384	0.4711	0.9686	0.8857	0.5352	0.7678
385	0.438	0.9636	0.876	0.4918	0.7401
386	0.4628	0.9537	0.8719	0.4853	0.7658
387	0.4959	0.9669	0.8884	0.5508	0.7728
388	0.405	0.9669	0.8733	0.4726	0.7345
389	0.4545	0.9603	0.876	0.4965	0.7777
390	0.5041	0.9686	0.8912	0.5626	0.7511

391	0.438	0.9554	0.8691	0.4682	0.7653
392	0.4545	0.957	0.8733	0.4872	0.7982
393	0.4876	0.9554	0.8774	0.5109	0.7894
394	0.4545	0.9537	0.8705	0.4782	0.7650
395	0.4545	0.962	0.8774	0.5013	0.7839
396	0.4545	0.9653	0.8802	0.5111	0.7245
397	0.4463	0.9669	0.8802	0.509	0.7796
398	0.4545	0.962	0.8774	0.5013	0.7884
399	0.438	0.9603	0.8733	0.4821	0.7664
400	0.4298	0.9636	0.8747	0.4846	0.7649
401	0.4545	0.9603	0.876	0.4965	0.7802
402	0.4463	0.9603	0.8747	0.4893	0.7773
403	0.4959	0.957	0.8802	0.5223	0.7502
404	0.438	0.9636	0.876	0.4918	0.7592
405	0.3967	0.9653	0.8705	0.46	0.7575
406	0.4545	0.9554	0.8719	0.4826	0.7289
407	0.4628	0.9587	0.876	0.4989	0.7814
408	0.4876	0.9587	0.8802	0.52	0.7865
409	0.4545	0.9669	0.8815	0.5161	0.7702
410	0.4876	0.9636	0.8843	0.5341	0.7897
411	0.4545	0.9719	0.8857	0.5317	0.7490
412	0.4876	0.962	0.8829	0.5293	0.7601
413	0.4628	0.9603	0.8774	0.5036	0.7804
414	0.438	0.9686	0.8802	0.507	0.7651
415	0.4711	0.962	0.8802	0.5154	0.7685
416	0.405	0.9686	0.8747	0.4778	0.7616
417	0.4463	0.9603	0.8747	0.4893	0.7606
418	0.4298	0.9653	0.876	0.4895	0.7841
419	0.4132	0.9669	0.8747	0.48	0.7655
420	0.4628	0.9686	0.8843	0.5282	0.7751
421	0.4545	0.9603	0.876	0.4965	0.7968
422	0.4793	0.9669	0.8857	0.5371	0.7641
423	0.4463	0.962	0.876	0.4941	0.7930
424	0.4545	0.962	0.8774	0.5013	0.7549
425	0.4545	0.9554	0.8719	0.4826	0.7511
426	0.4463	0.9603	0.8747	0.4893	0.7222
427	0.4215	0.9636	0.8733	0.4773	0.7527
428	0.4628	0.9587	0.876	0.4989	0.7685
429	0.4711	0.9636	0.8815	0.5202	0.7796

430	0.4463	0.962	0.876	0.4941	0.7577
431	0.4463	0.957	0.8719	0.48	0.7650
432	0.4793	0.9603	0.8802	0.5177	0.7565
433	0.4711	0.9603	0.8788	0.5107	0.7986
434	0.4628	0.9653	0.8815	0.5181	0.7661
435	0.4545	0.9636	0.8788	0.5061	0.7685
436	0.4545	0.9636	0.8788	0.5061	0.7564
437	0.4876	0.9587	0.8802	0.52	0.7899
438	0.4711	0.9587	0.8774	0.506	0.7586
439	0.4711	0.9669	0.8843	0.5301	0.7957
440	0.4132	0.9752	0.8815	0.507	0.7469
441	0.4545	0.9587	0.8747	0.4918	0.7591
442	0.4711	0.9636	0.8815	0.5202	0.7868
443	0.4711	0.9669	0.8843	0.5301	0.7386
444	0.4545	0.962	0.8774	0.5013	0.7720
445	0.5124	0.957	0.8829	0.536	0.7504
446	0.4876	0.957	0.8788	0.5154	0.7470
447	0.4463	0.957	0.8719	0.48	0.7600
448	0.4793	0.9669	0.8857	0.5371	0.7868
449	0.438	0.9653	0.8774	0.4968	0.7423
450	0.438	0.957	0.8705	0.4728	0.7261
451	0.4711	0.9587	0.8774	0.506	0.7555
452	0.4628	0.9603	0.8774	0.5036	0.7369
453	0.4711	0.962	0.8802	0.5154	0.8000
454	0.4711	0.962	0.8802	0.5154	0.7501
455	0.438	0.9636	0.876	0.4918	0.8025
456	0.4628	0.9702	0.8857	0.5334	0.7572
457	0.4793	0.9636	0.8829	0.5272	0.7573
458	0.4215	0.9587	0.8691	0.4628	0.7725
459	0.4793	0.9669	0.8857	0.5371	0.7383
460	0.4628	0.957	0.8747	0.4943	0.7905
461	0.4463	0.9636	0.8774	0.499	0.7900
462	0.4793	0.9554	0.876	0.5039	0.7624
463	0.5041	0.9554	0.8802	0.5247	0.7728
464	0.4545	0.9653	0.8802	0.5111	0.7590
465	0.4463	0.9653	0.8788	0.504	0.7788
